# Dynamical analysis for the motion of a 2DOF spring pendulum on a Lissajous curve

**DOI:** 10.1038/s41598-023-48523-5

**Published:** 2023-12-05

**Authors:** Asmaa Amer, T. S. Amer, H. F. El-Kafly

**Affiliations:** 1 Department of Mathematics and Computer Science, Faculty of Science, Menoufia University, Shebin El-Kom, Egypt; 2 Mathematics Department, Faculty of Science, Tanta University, Tanta, 31527 Egypt; 3 Tanta Higher Institute of Engineering and Technology, Tanta, Egypt

**Keywords:** Mathematics and computing, Applied mathematics

## Abstract

This study examines the motion of a spring pendulum with two degrees-of-freedom (DOF) in a plane as a vibrating system, in which its pivot point is constrained to move along a Lissajous curve. In light of the system’s coordinates, the governing equations of motion (EOM) are obtained utilizing the equations of Lagrange’s. The novelty of this work is to use the approach of multiple scales (AMS), as a traditional method, to obtain novel approximate solutions (AS) of the EOM with a higher degree of approximation. These solutions have been compared with the numerical ones that have been obtained using the fourth-order Runge–Kutta algorithm (4RKA) to reveal the accuracy of the analytic solutions. According to the requirements of solvability, the emergent resonance cases are grouped and the modulation equations (ME) are established. Therefore, the solutions at the steady-state case are confirmed. The stability/instability regions are inspected using Routh–Hurwitz criteria (RHC), and examined in accordance with the steady-state solutions. The achieved outcomes, resonance responses, and stability areas are demonstrated and graphically displayed, to evaluate the positive effects of different values of the physical parameters on the behavior of the examined system. Investigating zones of stability/instability reveals that the system’s behavior is stable for a significant portion of its parameters. A better knowledge of the vibrational movements that are closely related to resonance is crucial in many engineering applications because it enables the avoidance of on-going exposure to potentially harmful occurrences.

## Introduction

Undoubtedly, the act of inducing motion in dynamic systems, particularly those involving vibrations, plays a significant role in addressing various challenges encountered by researchers in the field of applied mechanics. This phenomenon can be replicated in certain machines, tools, mechanisms, or architectural structures through the application of external forces. The motion of big cars on highways and railroads, as well as vibrations brought on by earthquakes and the proximity of other machines, can also create similar excitations in machine supports. Many studies, including^1–3^, provide numerous examples of these excitations caused by rough rods, transverse waves, and sharp contact between the wheels of railroad trains and the track.

In Ref.^1^, it is investigated how a spring pendulum (SP) moves in relation to its two controllable factors, which are the energy and the frequencies of the spring and pendulum. Additionally, the authors investigated the phenomenon, specifically the back-and-forth movement of the spring and pendulum. By treating a long spring as a physical pendulum and formulating the mass in terms of the spring constant and various spring lengths, the estimation of the mass is considered necessary in Ref.^2^ to develop the resonance. For Reynolds number more than $10^{4}$, damped oscillations of SP model with a variable continuously diminishing mass are studied in Ref.^3^, in which the damping parameters are influenced by the mass loss rate.

In Refs.^4–9^ the dynamical behavior of a few various vibrational pendulum models connected with energy harvesting devices is examined as one of the best and most effective examples of converting mechanical energy into electric energy. The study conducted in Ref.^4^ investigates the vibrations of a two-degree-of-freedom spherical pendulum subjected to horizontal Lissajous excitation. By employing a mathematical model, the outcomes of numerical simulations are presented through visually appealing multi-colored maps, highlighting the behavior of the largest Lyapunov exponent. In a recent publication^5^, a groundbreaking design is presented, which encompasses a novel and sophisticated model of a double variable length cable pendulum. This model is accompanied by a meticulously designed experimental setup that incorporates elastic suspension and a counterweight mass for enhanced performance and accuracy. The investigation focuses on understanding the intricate dynamics that arise from the influence of varying lengths on the frequency and amplitude of vibrations. The study conducted in Ref.^6^ explores the interplay between parametric excitation and self-excited vibration within discontinuous systems. Through the use of a separate electromagnetic harvesting device, the pendulum’s structure is altered in Ref.^7^, where harvesting is dependent on the magnet in the coil oscillating. It is noted that the harvester’s effectiveness at both energy gathering and vibration reduction has increased. In Refs.^8,9^, 3DOF harvesting models have been examined. The model in Ref.^8^ is composed of two linked components: a nonlinear damping SP combined with an energy harvesting device and a nonlinear Duffing oscillator, while the other one in Ref.^9^ is formed up of two connected parts: the first is coupled to a piezoelectric transducer, which transforms stresses and oscillations into electrical power, whereas the second is a nonlinear damping SP.

A semi-analytical approach was used in Ref.^10^ to study the periodic movements of a periodically driven nonlinear SP, and the relevant stability and bifurcation analysis of these movements. After providing a consistent magnetic field in one direction, the motion of a SP is assessed in Ref.^11^. The AS, resembling Foucault’s pendulum, are also obtained when the heavy pendulum ball and delicate spring are taken into account.

The bifurcation phenomena at its state of equilibrium are discovered after examining how the magnetic field affects the stability of the SP. The implicit mapping approach is used in Ref.^12^ to calculate semi-analytically the entire bifurcation dynamics of period-3 motions to chaos. The harmonic amplitudes that change with excitation amplitudes to examine the complexity of periodic motion have been obtained. For the purpose of reducing vertical disturbances, a rotating pendulum absorber is suggested in Ref.^13^. By changing the rotating speed, the pendulum absorber’s characteristic frequency may be dynamically modified across a large range. The longitudinal and transverse absorbers that are linked with a SP are examined in Refs.^14–17^. The AMS^18^ is used to obtain the essential AS of the EOM, where the resonance situations are categorized and examined.

To evaluate the impact of the approximations of higher-order on the chaotic processes of a multi-DOF dynamical system with weak nonlinearity, Ref.^19^ examines a harmonically stimulated SP in a circular trajectory with internal resonance. A parametric and externally excited 2-DOF weakly nonlinearly system is investigated in Ref.^20^. There is a noticeable energy transfer between modes of vibration, where the selected resonance instance and the resonance conditions have been analysed and determined. The authors of Refs.^21,22^ build on the behavior of kinematically nonlinear excited SP, where its pivot point travels along an elliptic route. Simultaneous resonances have been studied in view of the exposed resonances circumstances.

In Ref.^23^, two different approaches have been used to resolve the motion of the nonlinear SP problem. According to the asymptotic analysis, three-time scales are utilized to get the AS with a respectable small error. In Ref.^24^, a generic model of a nonlinear damped excited SP is examined, in which its pivot point has been constrained to move along an ellipse trajectory with a stationary angular velocity. The AS are obtained up to the third correction. In Ref.^25^, the quadratic polynomial approximation was used to create an approximate controlling system with trigonometric nonlinearities. The presented unique approach ensures that the trigonometric functions are approximated with adequate precision not only around a specified point but also throughout the entire predetermined interval, contrary to the approximation accomplished using Taylor series. Thus, the suggested approximation is considered an approach that ensures better predictions for resonance responses in nonlinear mechanical systems. An approximation differential system is used to analyze the pendulum damper, which is modeled as a severely nonlinear auto-parametric system with 2DOF. As a foundation for the in-depth analytical analysis, the nature of the numerical solutions (NS) in the resonance state is examined. The resonant solution’s stationary and non-stationary properties are described in Ref.^26^. In Ref.^27^, the asymmetrical damping of a pendulum and its nonlinear properties, have been represented mathematically. Three distinct forms of the resonance domain were studied, and it was discovered that the excitation amplitude and the pendulum’s dynamic characteristics had a substantial impact on each type’s attributes.

In Ref.^28^, a few unusual states that can occur when a ball is moving in a sphere-shaped cavity acting as a passively tuned mass damper for thin engineered structures have been illustrated. Three non-holonomic restrictions are placed under horizontal additive kinematic excitation in a 6DOF system. The controlling differential system is determined using the Appell–Gibbs method^29^. In Ref.^30^, two viscous dampers and two linked nonlinear springs in series are used to analyze the forced planar vibrations of an attached particle at a supported point. The third-order correction law is proposed as the constitutive connection for the elastic forces of each spring. Three terms of Taylor series are used to simulate the resulting geometric nonlinearity from the pendulum’s transverse motion. In Refs.^31,32^, the frequency responses of a 2DOF nonlinear dynamical model that simulates the motion of a damped SP in an inviscid fluid flow are examined and discussed.

This work’s main objective is to investigate the motion of a 2DOF non-linearly damped SP system. It is assumed that two harmonically generated forces act in both the transverse and longitudinal directions, as well as a harmonic external moment at the pivot that restricts the pendulum motion to being on a Lissajous curve. The regulating EOM are derived applying Lagrange’s equations from the second type. For a higher level of accuracy, the EOM are analytically solved using AMS. The accuracy of the analytical solutions is determined by comparing them to the numerical ones that were derived using the 4RKA. In regard to the removal of secular factors, the solvability criteria and the ME are found. In order to confirm that the fixed points at steady-state solutions are stable or not, the RHC are applied. The non-linear stability analysis is used to expose various regions of stability or instability. A graphical examination of numerous plots associated with separate time-history curves, resonances, and stability zones is used to show how the system behaves. In many engineering applications, a deeper understanding of the vibrational motions that are closely associated with resonance is essential because it reduces the possibility of being continually exposed to potentially damaging events.

## Formulation of the dynamical system

**Figure 1:**
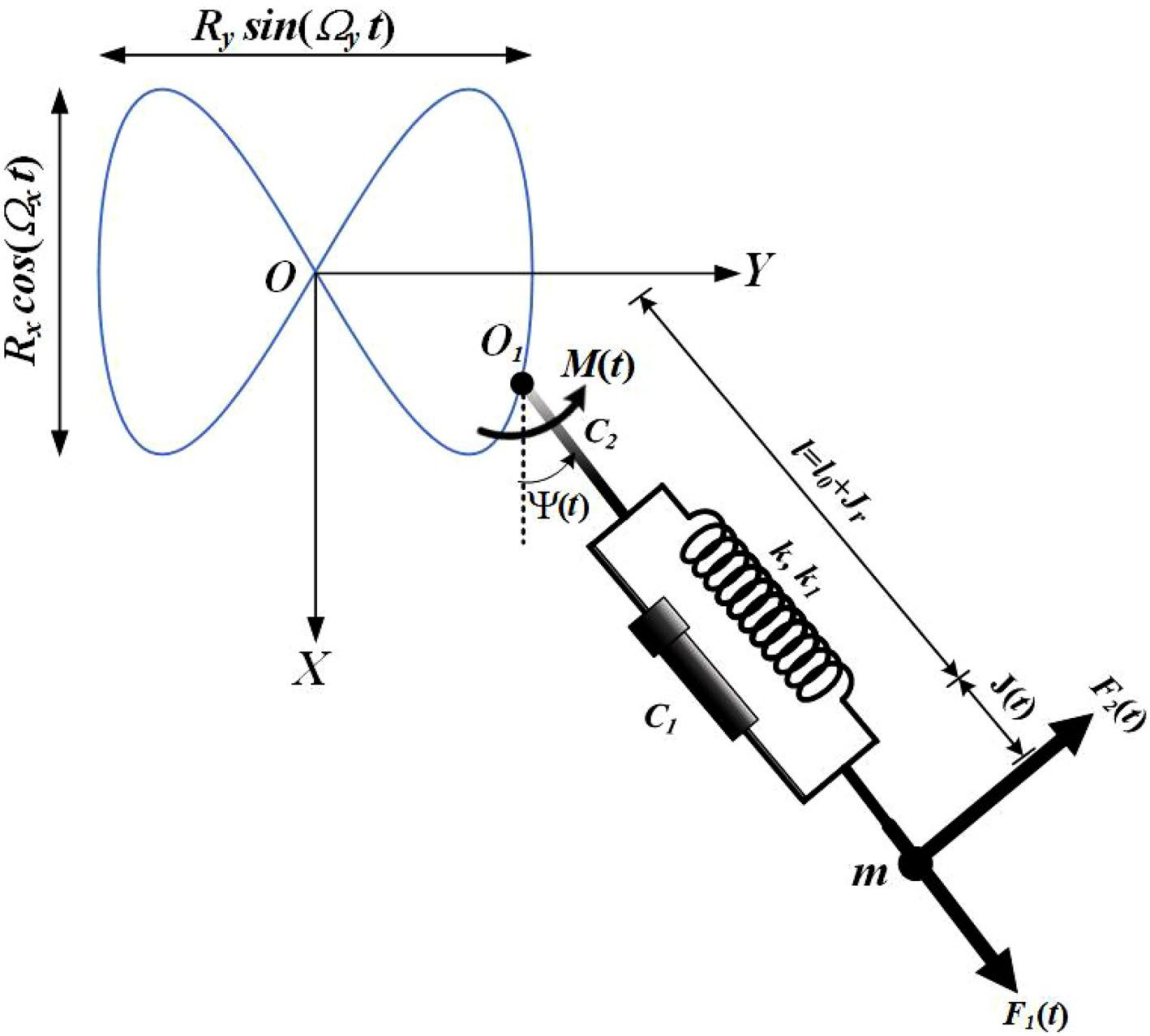
Dynamical sketch of the system.

The major objective of the present section is to derive the governing EOM of the examined dynamical system. This system consists of a non-linear damped SP with a massless normal length $l_{0}$ and stiffness $k$ and $k_{1}$. The suspension point $O_{1}$ of this pendulum is constrained to move on a route of a Lissajous curve in an anticlockwise direction, in which it moves independently and harmonically in two orthogonal directions, while the other point is connected with pendulum mass $m$ and oscillates in a plane. It is taken into account that point $O$, two orthogonal axes, $OX$ and $OY$, that are pointed, respectively, vertically and horizontally, are considered, see Fig. 1.

The coordinates $(x,y)$ describing the kinematic motion of the point $O_{1}$ are $x = R_{x} \cos \Omega_{x\,} t$ and $y = R_{y} \sin \Omega_{y\,} t$, where $R_{x} ,R_{y} ,\Omega_{x} ,$ and $\Omega_{y}$ represent known parameters. The planar motion of the investigated system is considered under the action of two perpendicular harmonic forces $F_{1} (t) = F_{1} \cos \Omega_{1} t$ and $F_{2} (t) = F_{2} \cos \Omega_{2} t$ in the radial and transverse directions of the spring, respectively, in addition to an anticlockwise moment $M(t) = M_{0} \cos \Omega_{0} t$ at $O_{1}$. Here, $F_{1} ,F_{2} ,M_{0}$ and $\Omega_{1} ,\Omega_{2} ,\Omega_{0}$ are amplitudes and frequencies of these forces and moment. Let $J(t)$ represents the elongation spring after time $t$, and $C_{j} \,\,(j = 1,2)$ are the viscous damping coefficients for longitudinal vibration and the swing one. These coefficients prevent the system from reaching a critical case in both vibration directions.

The following expression provides a foundation for formulating the system’s Lagrangian

1 $$ \begin{aligned} L = & \frac{1}{2}m{\kern 1pt} [R_{x}^{2} {\kern 1pt} \Omega_{x}^{2} {\kern 1pt} {\kern 1pt} \sin^{2} \Omega_{x} {\kern 1pt} t + R_{y}^{2} {\kern 1pt} \Omega_{y}^{2} \cos^{2} \Omega_{y} {\kern 1pt} t] + \frac{1}{2}m[\dot{J}^{2} + (l + J)^{2} \Psi^{2} ] \\ & + m{\kern 1pt} \dot{J}[R_{y} {\kern 1pt} \Omega_{y} {\kern 1pt} \sin \Psi \cos \Omega_{y} {\kern 1pt} t - R_{x} {\kern 1pt} \Omega_{x} {\kern 1pt} \cos \Psi \sin \Omega_{x} {\kern 1pt} t] \\ & + m{\kern 1pt} (l + J)\dot{\Psi }{\kern 1pt} [R_{y} {\kern 1pt} \Omega_{y} \cos \Psi \cos \Omega_{y} {\kern 1pt} t + R_{x} {\kern 1pt} \Omega_{x} \sin \Psi \sin \Omega_{x} {\kern 1pt} t] \\ & - \frac{1}{2}k(J + J_{r} )^{2} - \frac{1}{4}k_{1} (J + J_{r} )^{4} + mg[R_{x} \cos \Omega_{x} {\kern 1pt} t + (l + J)\cos \Psi ], \\ \end{aligned} $$

where $g$ denotes the acceleration of gravity, $\Psi$ denotes the swing’s angle, $l = l_{0} + J_{r} ,$ $J_{r} = {{mg} \mathord{\left/ {\vphantom {{mg} k}} \right. \kern-0pt} k}$ denotes the spring’s static elongation, and dots are the differentiation regards $t$. Equation (1) can subsequently be used to derive the governing EOM using the second type of Lagrange’s equations below

2 $$ \frac{d}{dt}\left( {\frac{\partial L}{{\partial \dot{q}}}} \right) - \frac{\partial L}{{\partial q}} = Q_{q} ;\,\,\,\,\,\,\,q( = J,\Psi ), $$

where $q$ stands for the system’s generalized coordinates and $Q_{q}$ represents a non-conservative generalized force that may be expressed as follows

3 $$ Q_{J} = F_{1} \cos \Omega_{1} {\kern 1pt} t - C_{1} {\kern 1pt} \dot{J}, $$

4 $$ Q_{\Psi } = (l + J)F_{2} \cos \Omega_{2} {\kern 1pt} t + M_{0} \cos \Omega_{0} {\kern 1pt} t - C_{2} \dot{\Psi }. $$

Consider the below dimensionless parameters

5 $$ \begin{gathered} \tau = \omega_{1} t,\,\,\,\,\,\,\,\,\Re = \frac{J}{l},\,\,\,\,\,\,\,\,\Re_{r} = \frac{{J_{r} }}{l},\,\,\,\,\,\,\,\,\omega_{1} = \sqrt{\frac{k}{m}}  ,\,\,\,\,\,\,\,\,\omega_{2} = \sqrt{\frac{g}{l}}  , \hfill \\ r_{x} = \frac{{R_{x} }}{l},\,\,\,\,\,\,\,\,r_{y} = \frac{{R_{y} }}{l},\,\,\,\,\,\,\,\,p_{x} = \frac{{\Omega_{x} }}{{\omega_{1} }},\,\,\,\,\,\,\,\,p_{y} = \frac{{\Omega_{y} }}{{\omega_{1} }},\,\,\,\,\,\,\,\,\alpha = \frac{{k_{1} l^{2} }}{{m{\kern 1pt} \omega_{1}^{2} }}, \hfill \\ c_{1} = \frac{{C_{1} }}{{m\omega_{1} }},\,\,\,\,\,\,\,\,c_{2} = \frac{{C_{2} }}{{ml^{2} \omega_{1} }},\,\,\,\,\,\,\,\,f_{1} = \frac{{F_{1} }}{{ml\omega_{1}^{2} }},\,\,\,\,\,\,\,\,f_{2} = \frac{{F_{2} }}{{ml\omega_{1}^{2} }}, \hfill \\ m_{0} = \frac{{M_{0} }}{{ml^{2} \omega_{1}^{2} }},\,\,\,\,\,\,\,\,p_{1} = \frac{{\Omega_{1} }}{{\omega_{1} }},\,\,\,\,\,\,\,\,p_{2} = \frac{{\Omega_{2} }}{{\omega_{1} }},\,\,\,\,\,\,\,\,p_{0} = \frac{{\Omega_{0} }}{{\omega_{1} }}\,\,\,\,\,\,\,\,\omega = \frac{{\omega_{2} }}{{\omega_{1} }}. \hfill \\ \end{gathered} $$

Based on the above Eqs. (1)–(5), one can obtain the desired dimensionless form of the EOM as follows

6 $$ \begin{aligned} \ddot{\Re } + & c_{1} \dot{\Re } + \Re + \alpha {\kern 1pt} \Re^{3} + 3\alpha {\kern 1pt} \Re_{r} {\kern 1pt} \Re^{2} + 3\alpha {\kern 1pt} \Re_{r}^{2} {\kern 1pt} \Re + \omega^{2} (1 - \cos \Psi ) - (1 + \Re )\dot{\Psi }^{2} \\ - & [r_{y} {\kern 1pt} p_{y}^{2} \sin \Psi \sin p_{y} {\kern 1pt} \tau + r_{x} {\kern 1pt} p_{x}^{2} \cos \Psi \cos p_{x} {\kern 1pt} \tau ] = f_{1} \cos p_{1} \tau , \\ \end{aligned} $$

7 $$ (1 + \Re )^{2} \ddot{\Psi } + c_{2} \dot{\Psi } + 2(1 + \Re )\dot{\Re }\dot{\Psi } + \omega^{2} (1 + \Re )\sin \Psi - (1 + \Re ){\kern 1pt} [r_{y} {\kern 1pt} p_{y}^{2} \cos \Psi \sin p_{y} {\kern 1pt} \tau - r_{x} {\kern 1pt} p_{x}^{2} \sin \Psi \cos p_{x} {\kern 1pt} \tau ] = (1 + \Re )f_{2} \cos p_{2} \tau + m_{0} \cos p_{0} {\kern 1pt} \tau , $$

where the dot denote the derivatives regarding $\tau$. The initial circumstances that constitute with the above EOM may be stated as follows pertaining

$$ \Re (0) = 0.04517369,\,\,\,\,\,\,\Psi (0) = 0.3029599,\,\,\,\,\,\,\dot{\Re }(0) = 0,\,\,\,\,\,\,\dot{\Psi }(0) = 0. $$

## The proposed method

In the current portion, the ASM can be used to achieve the solutions of the EOM (6) and (7) analytically, categorize the resonance situations, and get at the ME as well as the requirements of solvability. Therefore, we start by looking at the system’s vibrations in close proximity to its static equilibrium point^33^. Consequently, it is possible to approximate the trigonometric functions according to the following expressions

8 $$ \sin \Psi = \Psi - (\Psi^{3} /6),\,\,\,\,\,\,\,\,\,\,\,\,\,\,\,\,\cos \Psi = 1 - (\Psi^{2} /2). $$

Then, using a tiny parameter called $0 < \varepsilon < < 1$, we may represent the damping coefficients $c_{j} \,\,(j = 1,2)$, forces’ amplitudes $f_{j}$, moment’s amplitude $m_{0}$, and the parameters $r_{x} ,r_{y} ,\alpha$ as follows

9 $$ \begin{gathered} c_{j} = \varepsilon^{2} \,\tilde{c}_{j} ,\,\,\,\,\,\,f_{j} = \varepsilon^{3} \,\tilde{f}_{j} ,\,\,\,\,\,\,\,(j = 1,2) \hfill \\ m_{0} = \varepsilon^{3} \tilde{m}_{0} ,\,\,\,\,r_{x} = \varepsilon^{2} \,\tilde{r}_{x} \,,\,\,\,\,\,r_{y} = \varepsilon^{2} \,\tilde{r}_{y} ,\,\,\,\,\alpha = \varepsilon^{2} \tilde{\alpha }. \hfill \\ \end{gathered} $$

In a similar vein, we suppose that the vibrations’ amplitudes $\Re$ and $\Psi$ are of order $\varepsilon$. Then, one can write

10 $$ \Re (\tau ) = \varepsilon {\kern 1pt} \tilde{\Re }(\tau ;\varepsilon ),\,\,\,\,\Psi (\tau ) = \varepsilon \,\tilde{\Psi }(\tau ;\varepsilon ). $$

The expressions for the functions $\tilde{\Re }$ and $\tilde{\Psi }$ according to the AMS^18^ can be represented as follows

11 $$ \tilde{\Re }(\tau ,\varepsilon ) = \sum\limits_{s = 1}^{3} {\varepsilon^{s - 1} \tilde{\Re }_{s} (\tau_{0} ,} \tau_{1} ,\tau_{2} ) + O(\varepsilon^{3} ), $$

12 $$ \tilde{\Psi }(\tau ,\varepsilon ) = \sum\limits_{s = 1}^{3} {\varepsilon^{s - 1} \tilde{\Psi }_{s} (\tau_{0} ,} \tau_{1} ,\tau_{2} ) + O(\varepsilon^{3} ). $$

It is important to note that $\tau_{n} = \varepsilon^{n} \tau \,\,\,(n = 0,1,2)$ expresses new time scales that are reliant on $\tau$, where $\tau_{0}$ is rapid time scale whereas $\tau_{1}$ and $\tau_{2}$ are the slow ones. Additionally, due to their tiny size, terms of $O(\varepsilon^{2} )$ and higher orders have not been taken into account. To deal with the EOM (6) and (7) and the supposed solutions (11) and (12), we need to transform the time derivatives in (5) to be in relation to the time scales $\tau_{n}$, as follows

13 $$ \begin{aligned} \frac{d}{d\tau } &= \frac{\partial }{{\partial \tau_{0} }} + \varepsilon \frac{\partial }{{\partial \tau_{1} }} + \varepsilon^{2} \frac{\partial }{{\partial \tau_{2} }}, \hfill \\ \frac{{d^{2} }}{{d\tau^{2} }} &= \frac{{\partial^{2} }}{{\partial \tau_{0}^{2} }} + 2\varepsilon \frac{{\partial^{2} }}{{\partial \tau_{0} \partial \tau_{1} }} + \varepsilon^{2} \left( {\frac{{\partial^{2} }}{{\partial \tau_{1}^{2} }} + 2\frac{{\partial^{2} }}{{\partial \tau_{0} \partial \tau_{2} }}} \right). \hfill \\ \end{aligned} $$

Substituting (8) through (13) into (6) and (7) and equating coefficients of various powers of $\varepsilon$ with zero to obtain the below sets of partial differential equations (DEs).

Regarding order $$(\varepsilon )$$

14 $$ \begin{gathered} \frac{{\partial^{2} \tilde{\Re }_{1} }}{{\partial \tau_{0}^{2} }} + \tilde{\Re }_{1} = 0, \hfill \\ \frac{{\partial^{2} \tilde{\Psi }_{1} }}{{\partial \tau_{0}^{2} }} + \omega^{2} \tilde{\Psi }_{1} = 0. \hfill \\ \end{gathered} $$

Regarding order $$(\varepsilon^{2} )$$

15 $$ \begin{aligned} \frac{{\partial^{2} \tilde{\Re }_{2} }}{{\partial \tau_{0}^{2} }} + \tilde{\Re }_{2} &= \tilde{r}_{x} p_{x}^{2} \cos p_{x} {\kern 1pt} \tau_{0} - \frac{1}{2}\omega^{2} \tilde{\Psi }_{1}^{2} + (\frac{{\partial \tilde{\Psi }_{1} }}{{\partial \tau_{0} }})^{2} - 2\frac{{\partial^{2} \tilde{\Re }_{1} }}{{\partial \tau_{0} \partial \tau_{1} }}, \hfill \\ \frac{{\partial^{2} \tilde{\Psi }_{2} }}{{\partial \tau_{0}^{2} }} + \omega^{2} \tilde{\Psi }_{2} &= \tilde{r}_{y} p_{y}^{2} \sin p_{y} \tau_{0} - \omega^{2} \tilde{\Re }_{1} \tilde{\Psi }_{1} - 2\left( {\frac{{\partial \tilde{\Re }_{1} }}{{\partial \tau_{0} }}\frac{{\partial \tilde{\Psi }_{1} }}{{\partial \tau_{0} }} + \frac{{\partial^{2} \tilde{\Psi }_{1} }}{{\partial \tau_{0} \partial \tau_{1} }} + \tilde{\Re }_{1} \frac{{\partial^{2} \tilde{\Psi }_{1} }}{{\partial \tau_{0}^{2} }}} \right). \hfill \\ \end{aligned} $$

Regarding order $$(\varepsilon^{3} )$$

16 $$ \begin{aligned} \frac{{\partial^{2} \tilde{\Re }_{3} }}{{\partial \tau_{0}^{2} }} + \tilde{\Re }_{3} = & \tilde{f}_{1} \cos p_{1} \tau_{0} - \tilde{c}_{1} \frac{{\partial \tilde{\Re }_{1} }}{{\partial \tau_{0} }} - 3\tilde{\alpha }{\kern 1pt} \Re_{r}^{2} {\kern 1pt} \tilde{\Re }_{1} - \omega^{2} \tilde{\Psi }_{1} \tilde{\Psi }_{2} + \tilde{r}_{y} p_{y}^{2} \,\tilde{\Psi }_{1} \sin p_{y} {\kern 1pt} \tau_{0} - \frac{{\partial^{2} \tilde{\Re }_{1} }}{{\partial \tau_{1}^{2} }} \\ & + \tilde{\Re }_{1} \left( {\frac{{\partial \tilde{\Psi }_{1} }}{{\partial \tau_{0} }}} \right)^{2} - 2\left( {\frac{{\partial^{2} \tilde{\Re }_{1} }}{{\partial \tau_{0} \partial \tau_{2} }} + \frac{{\partial^{2} \tilde{\Re }_{2} }}{{\partial \tau_{0} \partial \tau_{1} }}} \right) + 2\frac{{\partial \tilde{\Psi }_{1} }}{{\partial \tau_{0} }}\left( {\frac{{\partial \tilde{\Psi }_{2} }}{{\partial \tau_{0} }} + \frac{{\partial \tilde{\Psi }_{1} }}{{\partial \tau_{1} }}} \right), \\ \frac{{\partial^{2} \tilde{\Psi }_{3} }}{{\partial \tau_{0}^{2} }} + \omega^{2} \tilde{\Psi }_{3} = & \tilde{f}_{2} \cos p_{2} \tau_{0} + \tilde{m}_{0} \cos p_{0} \tau_{0} + \tilde{r}_{y} p_{y}^{2} \,\tilde{\Re }_{1} \sin p_{y} {\kern 1pt} \tau_{0} - \tilde{r}_{x} p_{x}^{2} {\kern 1pt} \tilde{\Psi }_{1} \cos p_{x} {\kern 1pt} \tau_{0} \\ & - \tilde{c}_{2} \frac{{\partial \tilde{\Psi }_{1} }}{{\partial \tau_{0} }} - \omega^{2} \left( {\tilde{\Re }_{1} \tilde{\Psi }_{2} + \tilde{\Re }_{2} \tilde{\Psi }_{1} - \frac{{\tilde{\Psi }_{1}^{3} }}{6}} \right) - 2\frac{{\partial \tilde{\Psi }_{1} }}{{\partial \tau_{0} }}\left( {\tilde{\Re }_{1} \frac{{\partial \tilde{\Re }_{1} }}{{\partial \tau_{0} }} + \frac{{\partial \tilde{\Re }_{2} }}{{\partial \tau_{0} }} + \frac{{\partial \tilde{\Re }_{1} }}{{\partial \tau_{1} }}} \right) \\ & - \frac{{\partial^{2} \tilde{\Psi }_{1} }}{{\partial \tau_{1}^{2} }} - \frac{{\partial^{2} \tilde{\Psi }_{1} }}{{\partial \tau_{0}^{2} }}(\tilde{\Re }_{1}^{2} + 2\tilde{\Re }_{2} ) - 2\frac{{\partial \tilde{\Re }_{1} }}{{\partial \tau_{0} }}\left( {\frac{{\partial \tilde{\Psi }_{2} }}{{\partial \tau_{0} }} + \frac{{\partial \tilde{\Psi }_{1} }}{{\partial \tau_{1} }}} \right) - 2\tilde{\Re }_{1} \left( {\frac{{\partial^{2} \tilde{\Psi }_{2} }}{{\partial \tau_{0}^{2} }} + 2\frac{{\partial^{2} \tilde{\Psi }_{1} }}{{\partial \tau_{0} \partial \tau_{1} }}} \right) \\ & - 2\left( {\frac{{\partial^{2} \tilde{\Psi }_{2} }}{{\partial \tau_{0} \partial \tau_{1} }} + \frac{{\partial^{2} \tilde{\Psi }_{1} }}{{\partial \tau_{0} \partial \tau_{2} }}} \right). \\ \end{aligned} $$

The prior sets include six successively solvable second-order non-linear partial DEs, which emphasize the significance of the solutions of the first set (14). As a result, these equations’ generic solutions take the below forms

17 $$ \tilde{\Re }_{1} = D_{1} (\tau_{1} ,{\kern 1pt} \tau_{2} ){\kern 1pt} e^{{i\tau_{0} }} + \overline{D}_{1} (\tau_{1} ,{\kern 1pt} \tau_{2} ){\kern 1pt} e^{{ - i\tau_{0} }} , $$

18 $$ \tilde{\Psi }_{1} = D_{2} (\tau_{1} ,{\kern 1pt} \tau_{2} ){\kern 1pt} e^{{i\omega \tau_{0} }} + \overline{D}_{2} (\tau_{1} ,{\kern 1pt} \tau_{2} ){\kern 1pt} e^{{ - i\omega \tau_{0} }} , $$

where $i = \sqrt { - 1}$, $D_{j} \,\,(j = 1,\,2)$ are unknown dependent complex functions on $\tau_{j}$ and $\overline{D}_{j}$ are their complex conjugate.

Substituting the (17) and (18) into the second set of partial DEs (15) and removing the produced secular terms to obtain

19 $$ \frac{{\partial D_{1} }}{{\partial \tau_{1} }} = 0,\,\,\,\,\,\,\,\,\,\,\,\,\,\,\,\frac{{\partial D_{2} }}{{\partial \tau_{1} }} = 0. $$

Consequently, the second-order approximation can be expressed as follows.

20 $$ \tilde{\Re }_{2} = \omega^{2} D_{2} \overline{D}_{2} + \frac{{\tilde{r}_{x} p_{x}^{2} }}{{2{\kern 1pt} (1 - p_{x}^{2} )}}e^{{ip_{x} {\kern 1pt} \tau_{0} }} - \frac{{3\omega^{2} D_{2}^{2} }}{{2(1 - 4\omega^{2} )}}e^{{2i\omega \tau_{0} }} + {\text{c}}.{\text{c}}., $$

21 $$ \tilde{\Psi }_{2} = - \frac{{i{\kern 1pt} \tilde{r}_{y} {\kern 1pt} p_{y}^{2} }}{{2(\omega^{2} - p_{y}^{2} )}}e^{{i{\kern 1pt} p_{y} {\kern 1pt} \tau_{0} }} - \frac{{D_{1} \omega }}{{(4\omega^{2} - 1)}}\left[ {D_{2} (\omega + 2)(2\omega - 1)e^{{i(\omega + 1)\tau_{0} }} - \overline{D}_{2} (\omega - 2)(2\omega + 1)e^{{i(1 - \omega )\tau_{0} }} } \right] + c.c., $$

where the symbol $c.c.$ refers to the aforementioned terms’ complex conjugate.

To calculate the next requirements of solvability, substitute (17)–(21) into the third set of partial DEs (16) and then delete the apparent secular terms.

22 $$ \frac{{\partial D_{1} }}{{\partial \tau_{2} }} = \frac{i}{2}D_{1} \left[ {i\tilde{c}_{1} + 3\tilde{\alpha }{\kern 1pt} {\kern 1pt} \Re_{r}^{2} + \frac{{6\omega^{2} (\omega^{2} - 1)}}{{(4\omega^{2} - 1)}}D_{2} \overline{D}_{2} } \right], $$

23 $$ \frac{{\partial D_{2} }}{{\partial \tau_{2} }} = \frac{i}{4}D_{2} \left\{ {2i\tilde{c}_{2} + \frac{\omega }{{(4\omega^{2} - 1)}}[2(\omega^{2} + 2)(2\omega - 1)D_{1} \overline{D}_{1} + (3\omega^{2} + 1)(8\omega^{2} + 1)D_{2} \overline{D}_{2} ]} \right\}. $$

Consequently, the third-order approximation may be written as follows

24 $$ \tilde{\Re }_{3} = \frac{1}{4}\left\{ {\frac{{2\tilde{f}_{1} }}{{(1 - p_{1}^{2} )}}e^{{ip_{1} \tau_{0} }} + \frac{{2i{\kern 1pt} \tilde{r}_{y} {\kern 1pt} p_{y}^{3} (p_{y} + 2\omega )D_{2} }}{{(\omega^{2} - p_{y}^{2} )[1 - (\omega + p_{y} )^{2} ]}}e^{{i(\omega + p_{y} )\tau_{0} }} - \frac{{3\omega (\omega + 1)D_{1} D_{2}^{2} }}{(2\omega + 1)}e^{{i(2\omega + 1)\tau_{0} }} + \frac{{2i{\kern 1pt} \tilde{r}_{y} {\kern 1pt} p_{y}^{3} (p_{y} - 2\omega )\overline{D}_{2} }}{{(\omega^{2} - p_{y}^{2} )[1 - (p_{y} - \omega )^{2} ]}}e^{{i(p_{y} - \omega )\tau_{0} }} + \frac{{3\omega (\omega - 1)D_{1} \overline{D}_{2}^{2} }}{(2\omega - 1)}e^{{i(1 - 2\omega )\tau_{0} }} } \right\} + {\text{c}}.{\text{c}}., $$

25 $$ \begin{aligned} \tilde{\Psi }_{3} = & \frac{1}{2}\bigg\{ \frac{{\tilde{f}_{2} }}{{(\omega^{2} - p_{2}^{2} )}}e^{{ip_{2} \tau_{0} }} + \frac{{\tilde{m}_{0} }}{{(\omega^{2} - p_{0}^{2} )}}e^{{ip_{0} \tau_{0} }} - \frac{{i{\kern 1pt} \tilde{r}_{y} {\kern 1pt} p_{y}^{3} (p_{y} + 2)D_{1} }}{{{\kern 1pt} (\omega^{2} - p_{y}^{2} )[\omega^{2} - (p_{y} + 1)^{2} ]}}e^{{i(p_{y} + 1)\tau_{0} }} \\ & - \frac{{i{\kern 1pt} \tilde{r}_{y} {\kern 1pt} p_{y}^{3} (p_{y} - 2)\overline{D}_{1} }}{{(\omega^{2} - p_{y}^{2} )[\omega^{2} - (p_{y} - 1)^{2} ]}}e^{{i(p_{y} - 1)\tau_{0} }} + \frac{{\tilde{r}_{x} {\kern 1pt} p_{x} (p_{x}^{2} - 3\omega^{2} + 2\omega {\kern 1pt} p_{x} - 1)D_{2} }}{{(p_{x}^{2} - 1){\kern 1pt} (2\omega + p_{x} )}}e^{{i(p_{x} + \omega )\tau_{0} }} \\ & + \frac{{\tilde{r}_{x} {\kern 1pt} p_{x} {\kern 1pt} \left[ {(p_{x} - \omega )^{2} - 1} \right]\overline{D}_{2} }}{{(1 - p_{x}^{2} ){\kern 1pt} (2\omega - p_{x} )}}e^{{i(p_{x} - \omega )\tau_{0} }} + \frac{{\omega {\kern 1pt} (\omega + 2){\kern 1pt} (\omega + 3)D_{1}^{2} D_{2} }}{2(2\omega + 1)}e^{{i(\omega + 2)\tau_{0} }} \\ & + \frac{{\omega {\kern 1pt} (\omega - 2){\kern 1pt} (\omega - 3)D_{1}^{2} \overline{D}_{2} }}{2(2\omega - 1)}e^{{i(2 - \omega )\tau_{0} }} + \frac{{(1 - 13\omega^{2} )D_{2}^{3} }}{{24(4\omega^{2} - 1)}}e^{{3i\omega \tau_{0} }} \bigg\} + {\text{c}}.{\text{c}}. \\ \end{aligned} $$

The circumstances of eliminating secular terms (19), (22), and (23) can be used to calculate the functions $D_{j} \,\,(j = 1,\,2)$. One may readily find the asymptotic AS $\Re$ and $\Psi$ up to the third approximation according to the substitution of solutions [(11), (12)], [(17), (18)], [(20), (21)], and [(24), (25)] into (10).

Now, it’s important to highlight that the obtained AS remain acceptable when their dominators depart from zero^34^. However, resonant scenarios emerge when these dominators get closer to zero. As a result, one can categorise these scenarios as follows.

- The fundamental external resonance takes place at $$p_{1} = 1,$$$$p_{2} = \omega ,$$ and $$p_{0} = \omega$$.
- The internal resonance takes place at $p_{x} ( = 0,1,\,2{\kern 1pt} \omega ),$ $p_{y} = \omega ,$ and $\omega {\kern 1pt} {\kern 1pt} ( = 1,\, \pm 0.5)$.
- The combined resonances takes place at $p_{y} - \omega = \pm 1$ and $p_{y} + \omega = 1$.

It should be emphasized that if any of the preceding resonance scenarios occur, the behavior of the researched system would be difficult. Therefore, it would be necessary to alter the employed approach.

To address this issue, we will look into two fundamental external resonances that occur at the same time.

26 $$ p_{1} \approx 1,\,\,\,\,\,\,\,\,\,p_{2} \approx \omega . $$

These relationships demonstrate how closely $p_{1}$ to $1$ and $p_{2}$ to $\omega$. To achieve this purpose, it is important to provide dimensionless values $\sigma_{j} \,\,(j = 1,2)$ that are known by detuning parameters (which specify the separation between the strict resonance and vibrations) as follows

27 $$ p_{1} = 1 + \sigma_{1} ,\,\,\,\,\,\,\,\,\,\,\,p_{2} = \omega + \sigma_{2} . $$

In light of this, we can express $\sigma_{j}$ according to the use of $\varepsilon$ as

28 $$ \sigma_{j} = \varepsilon^{2} \tilde{\sigma }_{j} . $$

To obtain the following solvability requirements for the second and third-orders equations, substitute (27) and (28) into (15) and (16), and then eliminate terms that yield secular ones.

29 $$ \begin{array}{*{20}l} \frac{{\partial D_{1} }}{{\partial \tau_{1} }} = 0,\quad \frac{{\partial D_{2} }}{{\partial \tau_{1} }} = 0, \hfill \\ \frac{{\partial D_{1} }}{{\partial \tau_{2} }} = \frac{i}{4}\left\{ { - \tilde{f}_{1} e^{{i\tilde{\sigma }_{1} \tau_{2} }} + 2D_{1} [i\tilde{c}_{1} + 3\tilde{\alpha }{\kern 1pt} \Re_{r}^{2} {\kern 1pt} + \frac{{6\omega^{2} (\omega^{2} - 1)}}{{4\omega^{2} - 1}}D_{2} \overline{D}_{2} ]} \right\}, \hfill \\ \,\frac{{\partial D_{2} }}{{\partial \tau_{2} }} = \frac{i}{4}\left\{ { - \frac{{\tilde{f}_{2} }}{\omega }e^{{i\tilde{\sigma }_{2} \tau_{2} }} + 2D_{2} [i\tilde{c}_{2} + \frac{{\omega (\omega^{2} + 2)}}{2\omega + 1}D_{1} \overline{D}_{1} + \frac{{\omega (3\omega^{2} + 1)(8\omega^{2} + 1)}}{{2(4\omega^{2} - 1)}}D_{2} \overline{D}_{2} ]} \right\}. \hfill \\ \end{array} $$

A closer look at the aforementioned solvability requirements reveals that they combine to generate a system of four nonlinear partial DEs involving functions $D_{j} \,\,(j = 1,2)$. In addition, these functions are exclusively dependent on the slow time scale $\tau_{2}$, as explored in the first two requirements in (29). Hence, we provide $D_{j}$ in the polar form shown below

30 $$ D_{j} = \frac{{\tilde{a}_{j} (\tau_{2} )}}{2}e^{{i\psi_{j} (\tau_{2} )}} ,\,\,\,\,\,\,\,\,\,\,a_{j} = \varepsilon \tilde{a}_{j} , $$

where $\psi_{j} \,\,(j = 1,\,2)$ and $\tilde{a}_{j}$ are real functions that describe the phases and amplitudes of the solutions $\tilde{\Re }$ and $\tilde{\Psi }$.

The modelling procedures mentioned above show that the first-order derivatives of $D_{j}$ can be expressed as follows

31 $$ \partial D_{j} /\partial \tau = \varepsilon^{2} \partial D_{j} /\partial \tau_{2} . $$

In the context of (31), one can transform the partial DEs in (29) into ordinary DEs. Introducing (10), (13), (30), and (31), as well as the next adjusted phases

32 $$ \theta_{j} (\tau_{2} ) = \tilde{\sigma }_{j} {\kern 1pt} \tau_{2} - \psi_{j} (\tau_{2} );\,\,\,\,\,\,\,\,(j = 1,2), $$

into (29), distinguishing between real and imaginary portions to obtain the below autonomous system of four first-order ordinary DEs with regard to $a_{j}$ and $\theta_{j}$

33 $$ \frac{{da_{1} }}{d\tau } = \frac{1}{2}(f_{1} \sin \theta_{1} - c_{1} a_{1} ), $$

34 $$ \frac{{d\theta_{1} }}{d\tau } = \frac{1}{2}\left[ {\frac{{f_{1} }}{{a_{1} }}\cos \theta_{1} + 2\sigma_{1} - 3\alpha {\kern 1pt} \Re_{r}^{2} {\kern 1pt} + \frac{{3\omega^{2} (1 - \omega^{2} )}}{{2(4\omega^{2} - 1)}}a_{2}^{2} } \right], $$

35 $$ \frac{{da_{2} }}{d\tau } = \frac{1}{2}\left( {\frac{{f_{2} }}{\omega }\sin \theta_{2} - c_{2} {\kern 1pt} a_{2} } \right), $$

36 $$ \frac{{d\theta_{2} }}{d\tau } = \frac{1}{16}\left\{ {8\frac{{f_{2} }}{{\omega {\kern 1pt} a_{2} }}\cos \theta_{2} + 16\sigma_{2} - \frac{\omega }{{(4\omega^{2} - 1)}}[2(\omega^{2} + 2)(1 - 2\omega )a_{1}^{2} + (3\omega^{2} + 1)(8\omega^{2} + 1)a_{2}^{2} ]} \right\}. $$

**Figure 2:**
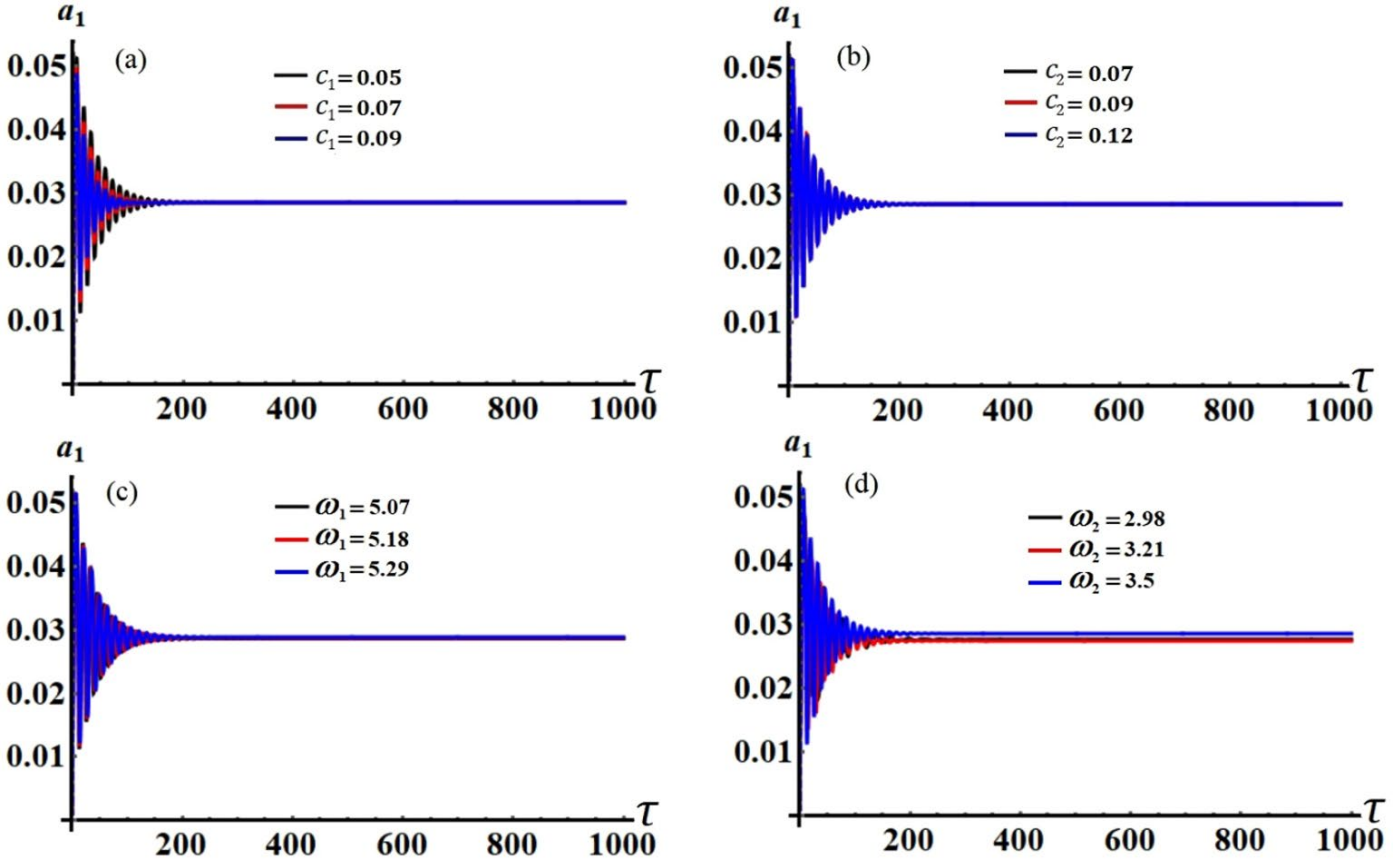
The temporal history of $a_{1}$ versus $\tau$: (**a**) when $c_{1} ( = 0.05,\,0.07,\,0.09),$ (**b**) when $c_{2} ( = 0.07,\,0.09,\,0.12),$ (**c**) when $\omega_{1} ( = 5.07,\,5.18,\,5.29),$ (**d**) when $\omega_{2} ( = 2.98,\,3.21,\,3.5)$.

**Figure 3:**
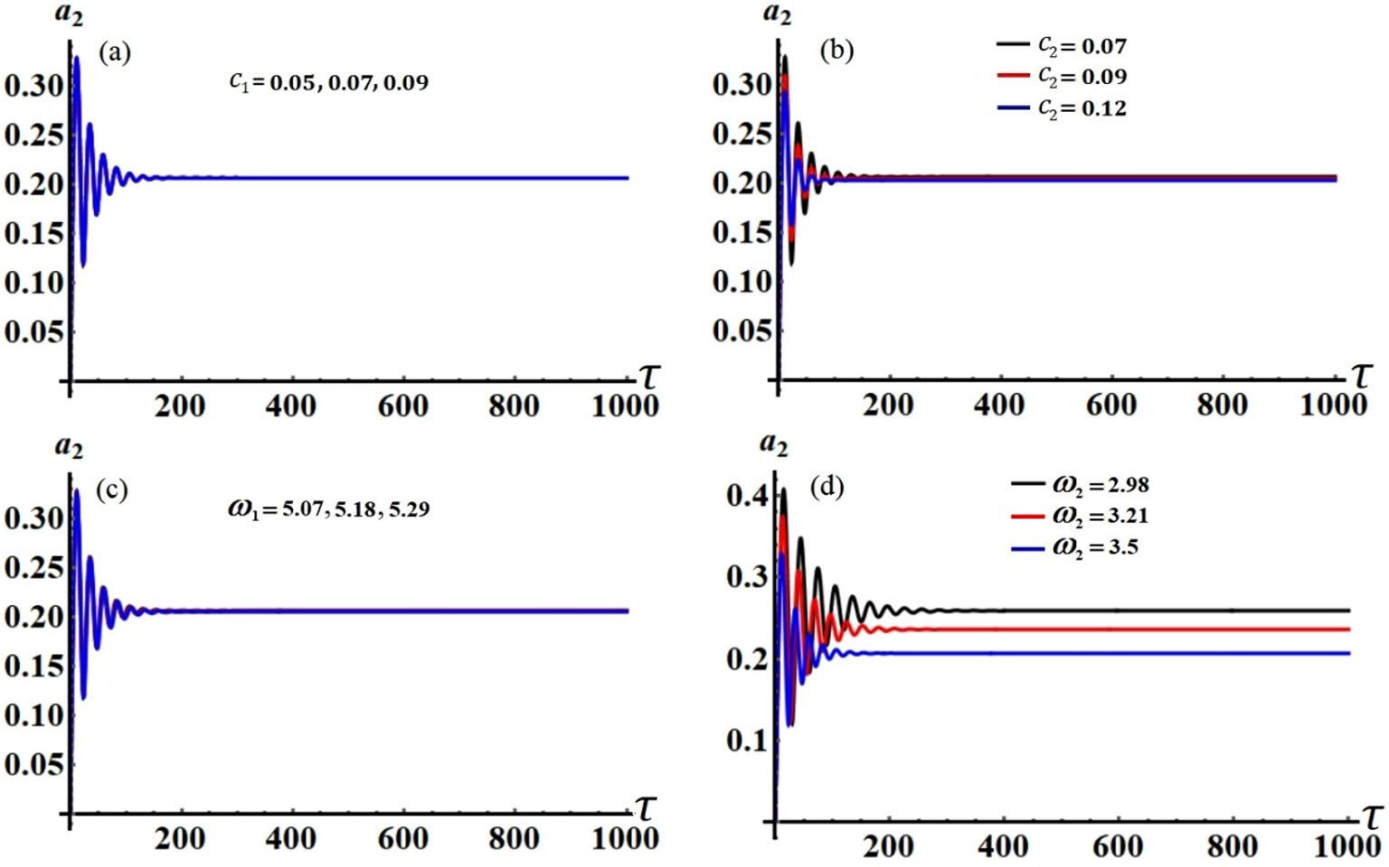
The behavior of $a_{2} (\tau )$: (**a**) when $c_{1} ( = 0.05,\,0.07,\,0.09),$ (**b**) when $c_{2} ( = 0.07,\,0.09,\,0.12),$ (**c**) when $\omega_{1} ( = 5.07,\,5.18,\,5.29),$ (**d**) when $\omega_{2} ( = 2.98,\,3.21,\,3.5)$**.**

**Figure 4:**
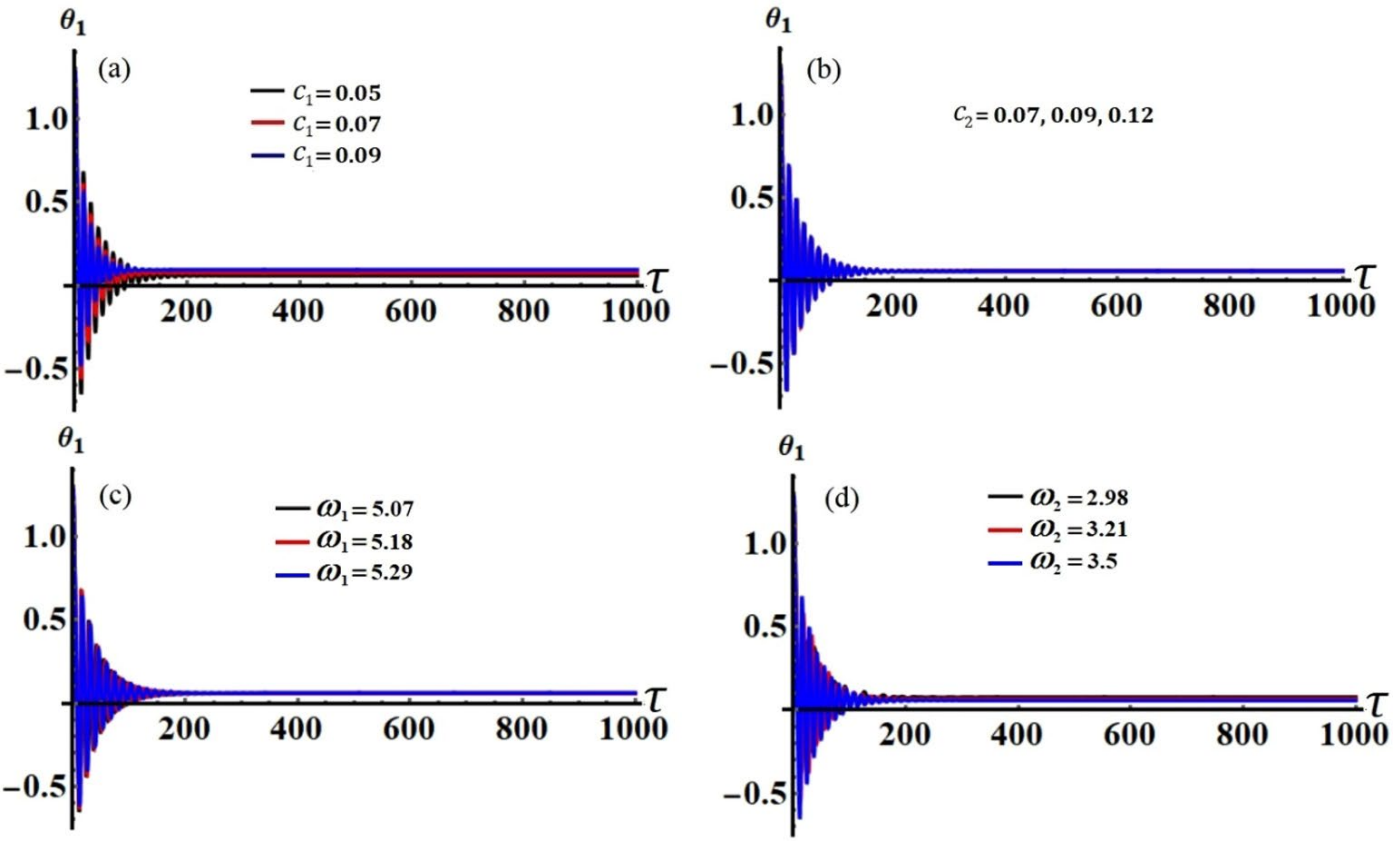
The variation of $\theta_{1} (\tau )$ during the time interval [0, 1000]: (**a**) when $c_{1} ( = 0.05,\,0.07,\,0.09),$ (**b**) when $c_{2} ( = 0.07,\,0.09,\,0.12),$ (**c**) when $\omega_{1} ( = 5.07,\,5.18,\,5.29),$ (**d**) when $\omega_{2} ( = 2.98,\,3.21,\,3.5)$**.**

**Figure 5:**
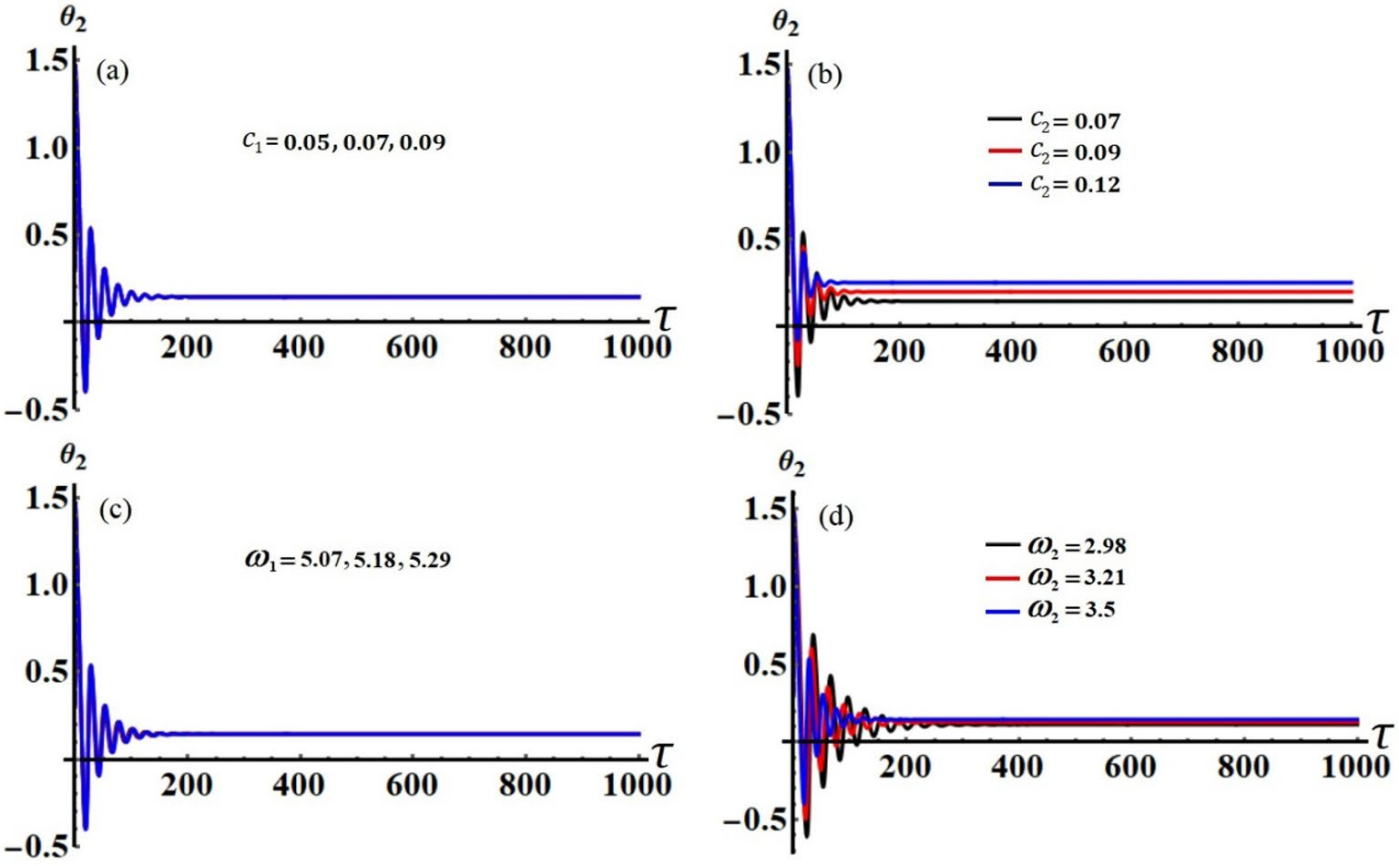
The variation of $\theta_{2}$ via $\tau$: (**a**) when $c_{1} ( = 0.05,\,0.07,\,0.09),$ (**b**) when $c_{2} ( = 0.07,\,0.09,\,0.12),$ (**c**) when $\omega_{1} ( = 5.07,\,5.18,\,5.29),$ (**d**) when $\omega_{2} ( = 2.98,\,3.21,\,3.5)$.

It is obvious that the aforementioned system of Eqs. (33)–(36) characterizes the ME for the two resonances that are being analysed concurrently. This system has been solved numerically to obtain the solutions $a_{j} (\tau )$ and $\theta_{j} (\tau )$, which are graphed in portions of Figs. 2, 3, 4 and 5 according to the following data of the used parameters

$$ \begin{gathered} R_{x} = 0.3,\quad R_{y} = 0.2,\,\,\,\,\,g = 9.8,\,\,\,\,\,l = 0.8,\quad m = 3.5,\quad \Omega_{0} = 2,\quad \Omega_{1} = 0.4,\quad \Omega_{2} = 2.4, \hfill \\ \Omega_{x} = 0.4,\quad \Omega_{y} = 0.5,\quad C_{1} = 1,\quad C_{2} = 0.8,\quad F_{1} = 2,\quad F_{2} = 5,\quad M_{0} = 0.5,\,\,\,\,\,\varepsilon = 0.0001, \hfill \\ k = 90,\,\,\,\,\,\,k_{1} = 30,\quad p_{1} = 1 + \sigma_{1} ,\,\,\,\,\,p_{2} = \omega + \sigma_{2} ,\quad c_{1} ( = 0.05,\,0.07,\,0.09), \hfill \\ c_{2} ( = 0.07,\,0.09,\,0.12),\quad \omega_{1} ( = 5.07,\,5.18,\,5.29),\quad \omega_{2} ( = 2.98,\,3.21,\,3.5). \hfill \\ \end{gathered} $$

Curves of these figures are calculated when the damping parameter $c_{1} ( = 0.05,\,0.07,\,0.09),$ $c_{2} ( = 0.07,\,0.09,\,0.12),$ and the frequencies $\omega_{1} ( = 5.07,\,5.18,\,5.29),$ $\omega_{2} ( = 2.98,\,3.21,\,3.5)$ have various values, as seen in potions (a), (b) and (c), (d) of these figures, respectively. These curves behave in a decaying manner, and they reach the stage of full stability at the end of the first fifth of the studied time interval when the aforementioned values are taken into account. It is noted that $a_{1}$ has been impacted by the change of the values of $c_{1}$ and $\omega_{2}$, as drawn, respectively, in Figs. 2a,d, 4a,d. In the same context, the temporal history of the amplitude $a_{2}$ is influenced by the change of the values $c_{2}$ and $\omega_{2}$, as seen, respectively, in Figs. 3b,d, 5b,d. A closer look at the other parts of Figs. 2, 3, 4 and 5, one can observe that they haven’t any variation with the change of $c_{j}$ and $\omega_{j}$. The reason for the change or non-change is due to the mathematical composition of the equations of system (33)–(36), as the first and third equations are dependent on $c_{1}$ and $\omega_{2}$, respectively. Whereas they do not explicitly depend on the variable $c_{2}$ and $\omega_{1}$. Similarly, the second and fourth equations of the same system is independent on $c_{1}$ and $\omega_{1}$, in which they are depend on $c_{2}$ and $\omega_{2}$.

**Figure 6:**
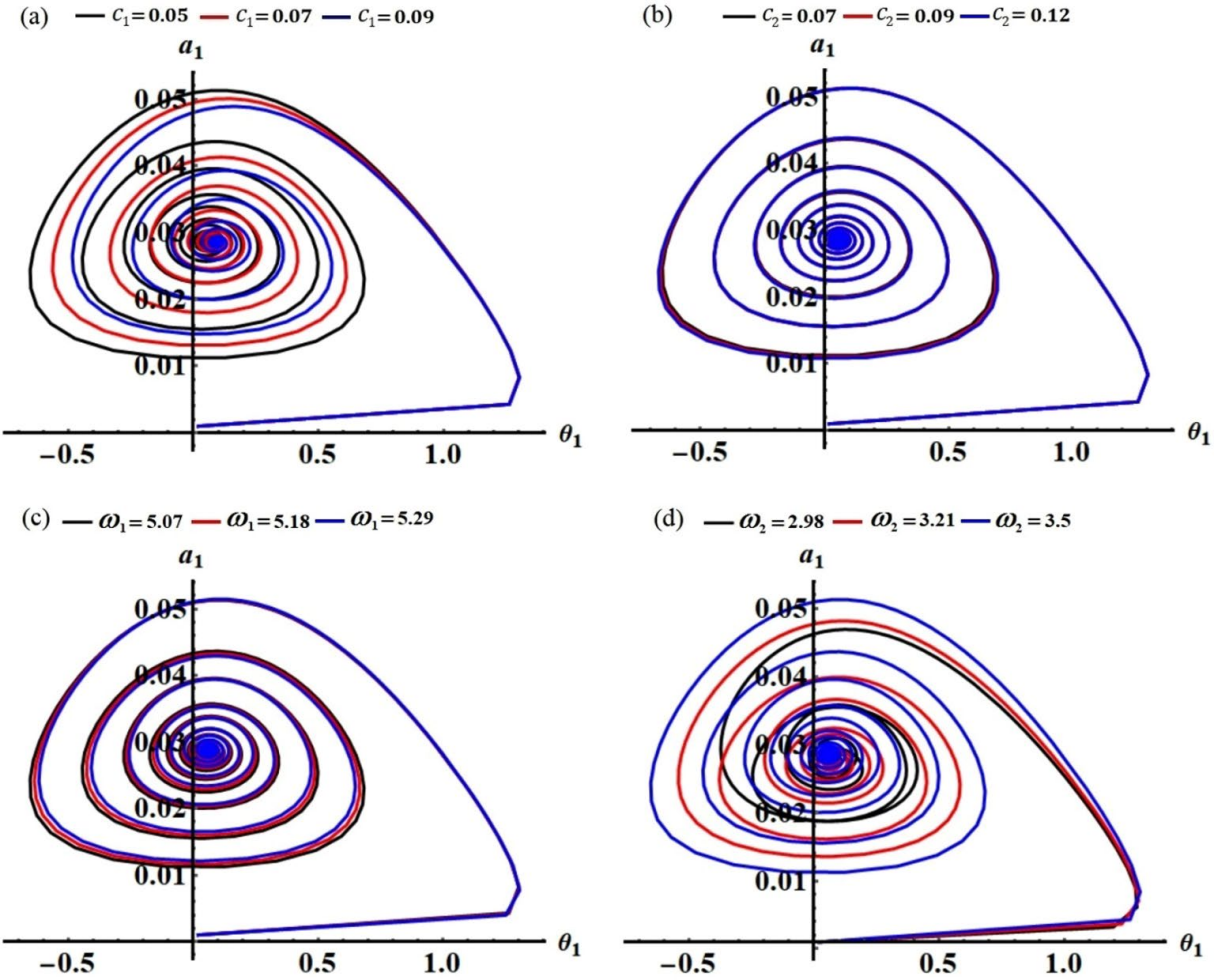
The curves in the plane $\theta_{1} a_{1}$ at: (**a**) $c_{1} ( = 0.05,\,0.07,\,0.09),$ (**b**) $c_{2} ( = 0.07,\,0.09,\,0.12),$ (**c**) $\omega_{1} ( = 5.07,\,5.18,\,5.29),$ (**d**) $\omega_{2} ( = 2.98,\,3.21,\,3.5)$.

**Figure 7:**
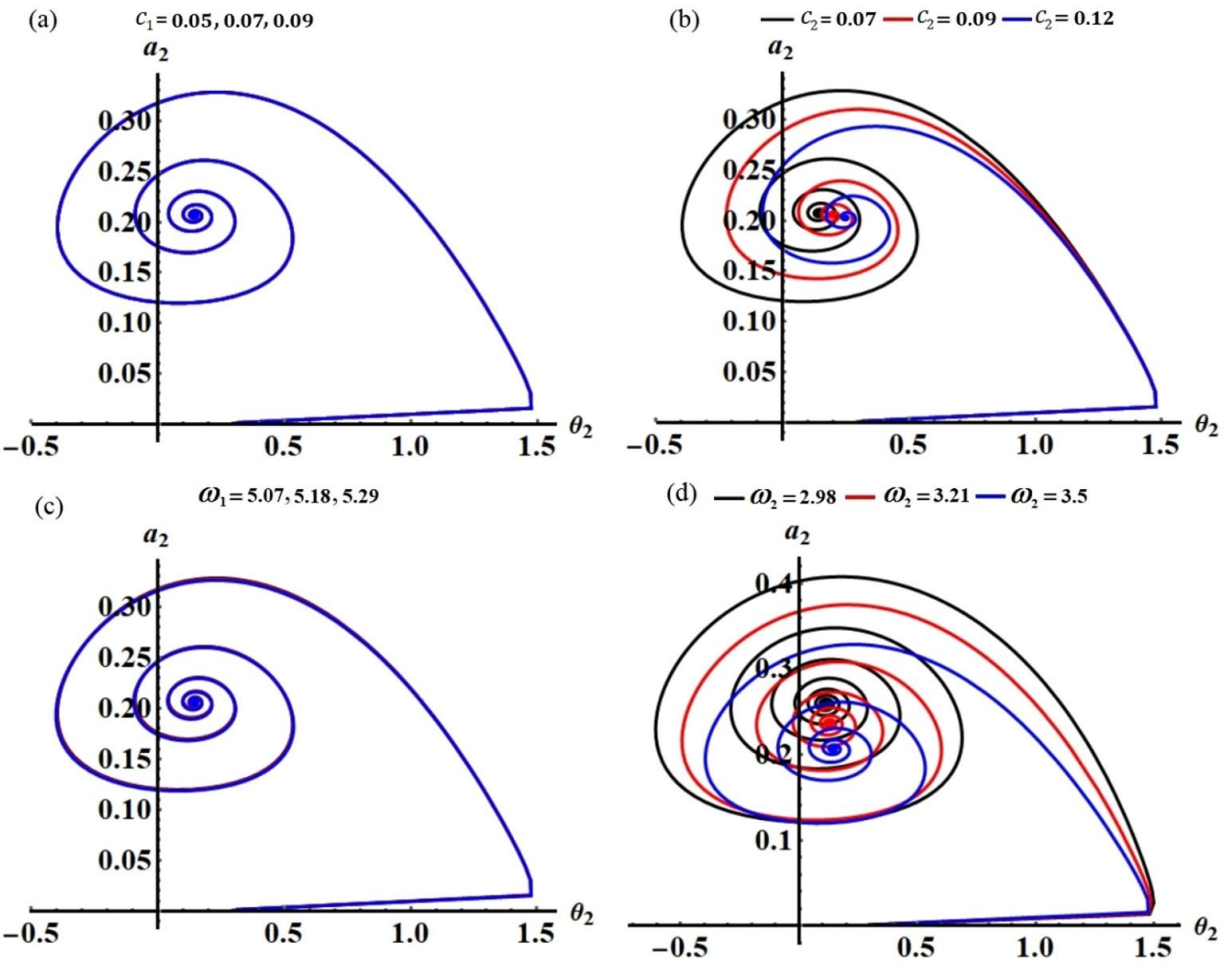
The curves in the plane $\theta_{2} a_{2}$ at: (**a**) $c_{1} ( = 0.05,\,0.07,\,0.09),$ (**b**) $c_{2} ( = 0.07,\,0.09,\,0.12),$ (**c**) $\omega_{1} ( = 5.07,\,5.18,\,5.29),$ (**d**) $\omega_{2} ( = 2.98,\,3.21,\,3.5)$.

The projections of the plotted curves in Figs. 2, 3, 4 and 5 in the planes $\theta_{1} a_{1}$ and $\theta_{2} a_{2}$ are drawn in portions of Figs. 6 and 7. The behaviors of these curves have the form of spiral curves that are directed towards one point, which means that the functions described by these curves are stable. This conclusion is consistent with the above discussion of Figs. 2, 3, 4 and 5 and with the equations of system (33)–(36). It must be emphasized that the changes that occurred in the curves drawn in Figs. 2, 3, 4 and 5 correspond to similar changes in Figs. 6 and 7 at the same values of the different parameters, whose effect on the behaviors of the waves has been studied.

**Figure 8:**
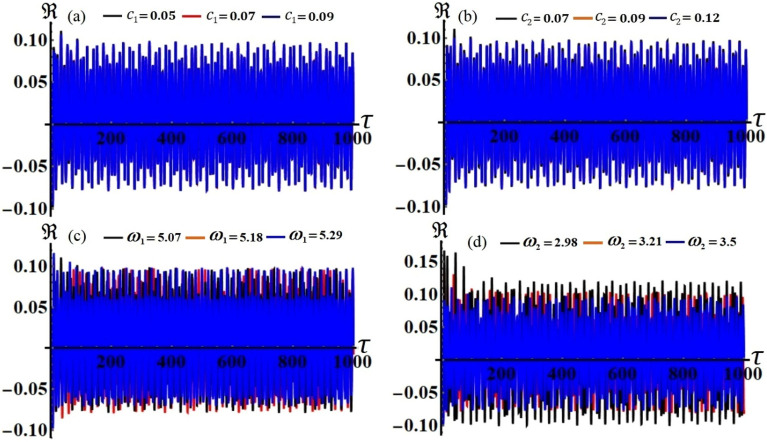
The behavior of the spring’s elongation $\Re (\tau )$ at: (**a**) $c_{1} ( = 0.05,\,0.07,\,0.09),$ (**b**) $c_{2} ( = 0.07,\,0.09,\,0.12),$ (**c**) $\omega_{1} ( = 5.07,\,5.18,\,5.29),$ (**d**) $\omega_{2} ( = 2.98,\,3.21,\,3.5)$.

**Figure 9:**
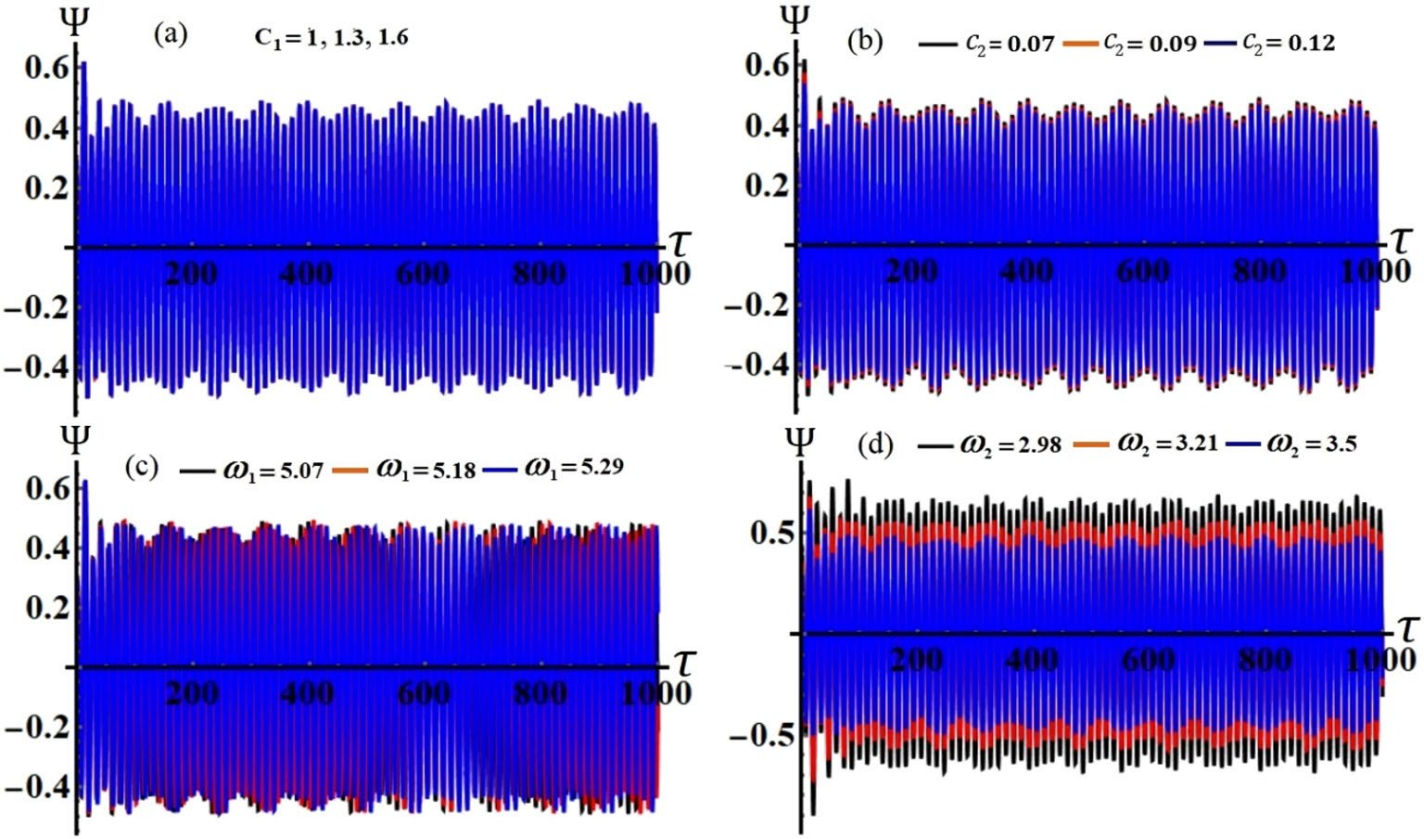
The behavior of the rotation angle $\Psi (\tau )$ at: (**a**) $c_{1} ( = 0.05,\,0.07,\,0.09),$ (**b**) $c_{2} ( = 0.07,\,0.09,\,0.12),$ (**c**) $\omega_{1} ( = 5.07,\,5.18,\,5.29),$ (**d**) $\omega_{2} ( = 2.98,\,3.21,\,3.5)$.

**Figure 10:**
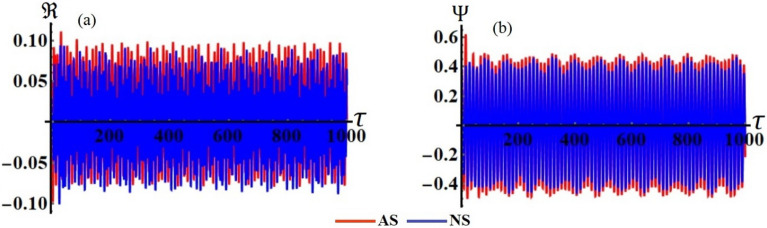
The comparison between the AS and NS at $c_{1} = 0.05,$ $c_{2} = 0.07,$ $\,\omega_{1} = 5.07,$ and $\omega_{2} = 3.5$ for the: (**a**) solution $\Re (\tau )$, and (**b**) solution $\Psi (\tau )$.

Figures 8 and 9 present, respectively, the achieved analytical solutions $\Re (\tau )$ and $\Psi (\tau )$ to highlight the temporal behavior of these solutions while taking into account the prior values of the system’s parameters. This behavior has the form of quasi-periodic waves. It must be mentioned that these have been impacted more by the various values of the frequencies $\omega_{1}$ and $\omega_{2}$ than the damping parameters $c_{1}$ and $c_{2}$. The accuracy of the analytical solutions is evaluated by comparing them to the numerical ones of the original EOM that were produced using 4RKA according to the plotted curves in Fig. 10 at $c_{1} = 0.05,$ $c_{2} = 0.07,$ $\,\omega_{1} = 5.07,$ and $\omega_{2} = 3.5$. The comparison demonstrates excellent agreement between both solutions.

## Solutions at the scenario of steady-state

The purpose of the current section is to investigate the steady-state oscillations of the considered dynamical system. In essence, temporary processes will cease to exist under the impact of damping, and the steady-state oscillations will then be generated^35–38^. Therefore, we regard the left-hand side of the system of ME (33)–(36) as zero. As a consequence, the equations of this system have been transformed into the algebraic equations shown below

37 $$ c_{1} a_{1} - f_{1} \sin \theta_{1} = 0, $$

38 $$ \frac{{f_{1} }}{{a_{1} }}\cos \theta_{1} + 2\sigma_{1} - 3\alpha {\kern 1pt} \Re_{r}^{2} {\kern 1pt} + \frac{{3\omega^{2} (1 - \omega^{2} )}}{{2(4\omega^{2} - 1)}}a_{2}^{2} = 0, $$

39 $$ \omega c_{2} a_{2} - f_{2} \sin \theta_{2} = 0, $$

40 $$ 8\frac{{f_{2} }}{{\omega {\kern 1pt} a_{2} }}\cos \theta_{2} + 16\sigma_{2} - \frac{\omega }{{(4\omega^{2} - 1)}}[2(\omega^{2} + 2)(1 - 2\omega )a_{1}^{2} + (3\omega^{2} + 1)(8\omega^{2} + 1)a_{2}^{2} ] = 0. $$

After removing the adjusted phases $\theta_{j} \,\,\,(j = 1,2)$ from the system of Eqs. (37)–(40), the next nonlinear algebraic equations regarding the parameters of detuning $\sigma_{j}$ and adjusted amplitudes $a_{j}$ are obtained.

41 $$ \begin{array}{*{20}l} f_{1}^{2} = a_{1}^{2} \left\{ {c_{1}^{2} + \left[ {2\sigma_{1} - 3\alpha \,\Re_{r}^{2} + \frac{{3\omega^{2} (1 - \omega^{2} )}}{{2(4\omega^{2} - 1)}}a_{2}^{2} } \right]^{2} } \right\}, \hfill \\ f_{2}^{2} = \omega^{2} a_{2}^{2} \left\{ {c_{2}^{2} + \left[ {2\sigma_{2} - \frac{{\omega (\omega^{2} + 2)}}{4(2\omega + 1)}a_{1}^{2} - \frac{{\omega (3\omega^{2} + 1)(8\omega^{2} + 1)}}{{8(4\omega^{2} - 1)}}a_{2}^{2} } \right]^{2} } \right\}. \hfill \\ \end{array} $$

One of the most important aspects of steady-state oscillations is to analyse the stability. To analyse such a scenario, the system’s behavior will be evaluated in a relatively close neighbourhood area to the locations of fixed points. To accomplish this purpose, we consider the next substitutions in (33)–(36)

42 $$ \begin{gathered} a_{1} = a_{10} + a_{11} ,\,\,\,\,\,\,\,\,\,\,\,\,\,\,\,\theta_{1} = \theta_{10} + \theta_{11} , \hfill \\ a_{2} = a_{20} + a_{21} ,\,\,\,\,\,\,\,\,\,\,\,\,\,\,\theta_{2} = \theta_{20} + \theta_{21} , \hfill \\ \end{gathered} $$

where $a_{j0} \,\,(j = 1,2)$ and $\theta_{j0}$ denote the solutions at the steady-state, while $a_{j1}$ and $\theta_{j1}$ denote relatively minor disturbances in comparison to $a_{j0}$ and $\theta_{j0}$. Consequently, after linearization, one gets

43 $$ \frac{{da_{11} }}{d\tau } = \frac{1}{2}(f_{1} {\kern 1pt} \theta_{11} \cos \theta_{10} - c_{1} a_{11} ), $$

44 $$ a_{10} \frac{{d\theta_{11} }}{d\tau } = \frac{1}{4}\left[ {2a_{11} (2\sigma_{1} - 3\alpha \,\Re_{r}^{2} ) + \frac{{3\omega^{2} (1 - \omega^{2} )}}{{(4\omega^{2} - 1)}}a_{20} (a_{11} a_{20} + 2a_{10} a_{21} ) - 2f_{1} \theta_{11} \sin \theta_{10} } \right], $$

45 $$ \frac{{da_{21} }}{d\tau } = \frac{1}{2}\left( {\frac{{f_{2} }}{\omega }\theta_{21} \cos \theta_{20} - c_{2} a_{21} } \right), $$

46 $$ a_{20} \frac{{d\theta_{21} }}{d\tau } = \frac{1}{16}\left\{ {a_{21} \left[ {16\sigma_{2} - \frac{{3\omega (3\omega^{2} + 1)(8\omega^{2} + 1)}}{{(4\omega^{2} - 1)}}a_{20}^{2} } \right] - \frac{{2\omega (\omega^{2} + 2)}}{(2\omega + 1)}a_{10} (a_{10} a_{21} + 2a_{20} a_{11} ) - \frac{{8f_{2} }}{\omega }\theta_{21} \sin \theta_{20} } \right\}. $$

Remembering that $a_{j1}$ and $\theta_{j1}$ are defined, respectively, above as unknown perturbed functions of amplitudes and phases in the preceding system. Then we are able to outline their solutions as a linear superposition of $q_{s} e^{\lambda \tau } \,\,(s = 1,2,3,4)$, where $q_{s}$ represent constants and $\lambda$ expresses the eigenvalue of these functions. If the solutions $a_{j0}$ and $\theta_{j0}$ are stable asymptotically, then the real components of the roots of the yielded characteristic equation of the system (43)–(46) must be negative

47 $$ \lambda^{4} + \Gamma_{1} \lambda^{3} + \Gamma_{2} \lambda^{2} + \Gamma_{3} \lambda + \Gamma_{4} = 0, $$

where

$$ \begin{aligned} \Gamma_{1} = & \frac{1}{{2{\kern 1pt} }}\left( {c_{1} + c_{2} + \frac{{f_{1} \sin \theta_{10} }}{{a_{10} }} + \frac{{f_{2} \sin \theta_{20} }}{{\omega {\kern 1pt} a_{20} }}} \right), \\ \Gamma_{2} = & \frac{1}{{32{\kern 1pt} {\kern 1pt} \omega {\kern 1pt} {\kern 1pt} (4\omega^{2} - 1)a_{10} a_{20} }}\{ 8(4\omega^{2} - 1)f_{2} \sin \theta_{20} [a_{10} (c_{1} + c_{2} ) + f_{1} \sin \theta_{10} ] \\ & + 4\omega {\kern 1pt} a_{20} \{ 2{\kern 1pt} (4\omega^{2} - 1)a_{10} {\kern 1pt} {\kern 1pt} c_{1} c_{2} + f_{1} [3{\kern 1pt} \omega^{2} (\omega^{2} - 1)\cos \theta_{10} {\kern 1pt} a_{20}^{2} \\ & + 2(4\omega^{2} - 1)[(c_{1} + c_{2} )\sin \theta_{10} + (3\alpha {\kern 1pt} \Re_{r}^{2} - 2\sigma_{1} )\cos \theta_{10} ]]\} \\ & + a_{10} {\kern 1pt} f_{2} \cos \theta_{20} [2\omega (2\omega - 1)(\omega^{2} + 2)a_{10}^{2} + 3\omega (3\omega^{2} + 1)(8\omega^{2} + 1)a_{20}^{2} \\ & + 16\sigma_{2} (1 - 4\omega^{2} )]\} , \\ \end{aligned} $$

$$ \begin{aligned} \Gamma_{3} = & \frac{1}{{64{\kern 1pt} \omega {\kern 1pt} (4\omega^{2} - 1){\kern 1pt} a_{10} {\kern 1pt} a_{20} }}\{ 4f_{2} \sin \theta_{20} \{ 2(4\omega^{2} - 1)a_{10} c_{1} c_{2} + f_{1} [3\omega^{2} (\omega^{2} - 1){\kern 1pt} a_{20}^{2} {\kern 1pt} \cos \theta_{10} \\ & + 2(4\omega^{2} - 1)[(c_{1} + c_{2} )\sin \theta_{10} + (3\alpha {\kern 1pt} \Re_{r}^{2} - 2\sigma_{1} )\cos \theta_{10} ]]\} + 4{\kern 1pt} c_{2} {\kern 1pt} a_{20} f_{1} \{ 3a_{20}^{2} \omega^{3} (\omega^{2} - 1)\cos \theta_{10} \\ & + 2\omega (4\omega^{2} - 1)[c_{1} \sin \theta_{10} + (3\alpha {\kern 1pt} \Re_{r}^{2} - 2\sigma_{1} )\cos \theta_{10} ]\} + f_{2} \cos \theta_{20} (a_{10} c_{1} \\ & + f_{1} \sin \theta_{10} )[2\omega (2\omega - 1)(\omega^{2} + 2)a_{10}^{2} + 3\omega (3\omega^{2} + 1)(8\omega^{2} + 1)a_{20}^{2} + 16\sigma_{2} (1 - 4\omega^{2} ){\kern 1pt} ]\} , \\ \end{aligned} $$

48 $$ \begin{aligned} \Gamma_{4} = & \frac{{f_{1} f_{2} }}{{256\omega (1 - 4\omega^{2} )^{2} (2\omega^{2} - 1)a_{10} a_{20} }}\{ - 2\omega (2\omega - 1)(\omega^{2} + 2)\cos \theta_{20} a_{10}^{2} [3\omega^{2} \\ & \times (14\omega^{4} - 17\omega^{2} + 3)a_{20}^{2} \cos \theta_{10} - 2(8\omega^{4} - 6\omega^{2} + 1)[c_{1} \sin \theta_{10} + (3\alpha {\kern 1pt} \Re_{r}^{2} - 2\sigma_{1} )\cos \theta_{10} ]] \\ & + (2\omega^{2} - 1)\{ [3\omega^{2} (\omega^{2} - 1)a_{20}^{2} \cos \theta_{10} + 2(4\omega^{2} - 1)[c_{1} \sin \theta_{10} + (3\alpha {\kern 1pt} \Re_{r}^{2} - 2\sigma_{1} )\cos \theta_{10} ]] \\ & \times [3\omega (24\omega^{4} + 11\omega^{2} + 1)a_{20}^{2} \cos \theta_{20} + 8(4\omega^{2} - 1)[c_{2} \sin \theta_{20} - 2\sigma_{2} \cos \theta_{20} ]]\} \} . \\ \end{aligned} $$

To determine the requisite stability criteria for the solutions in a certain steady state, the following RHC^18^ can be used

49 $$ \begin{array}{*{20}l} \Gamma_{1} > 0,\,\,\,\,\,\,\,\,\,\Gamma_{3} (\Gamma_{1} \Gamma_{2} - \Gamma_{3} ) - \Gamma_{4} \Gamma_{1}^{2} > 0 \hfill \\ \Gamma_{1} \Gamma_{2} - \Gamma_{3} > 0,\,\,\,\,\,\,\,\,\,\Gamma_{4} > 0. \hfill \\ \end{array} $$

## The stability analysis

This section explores the stability of the examined system using the non-linear stability approach of Routh–Hurwitz. It must be remembered that the system under consideration consists of a moving, nonlinear, damped spring pendulum in a Lissajous route, which is influenced by an external harmonic moment $M(t)$ as well as two perpendicular forces $F_{1} (t)$ and $F_{2} (t)$. The requirements of stability are applied alongside the simulation of the system’s non-linear evolution. A number of variables, such as damping coefficients $c_{j} \,\,(j = 1,2)$, frequencies $\omega_{j}$, and detuning parameters $\sigma_{j}$, have been discovered to have a vital influence in the stability criteria.

The stability diagrams of the system of Eqs. (33)–(36) are obtained by performing certain actions with different parameters of the system. The variation of adjusted amplitudes $a_{j}$ with time is plotted for different parametrical zones, and its characteristics are presented using phase plane paths.

**Figure 11:**
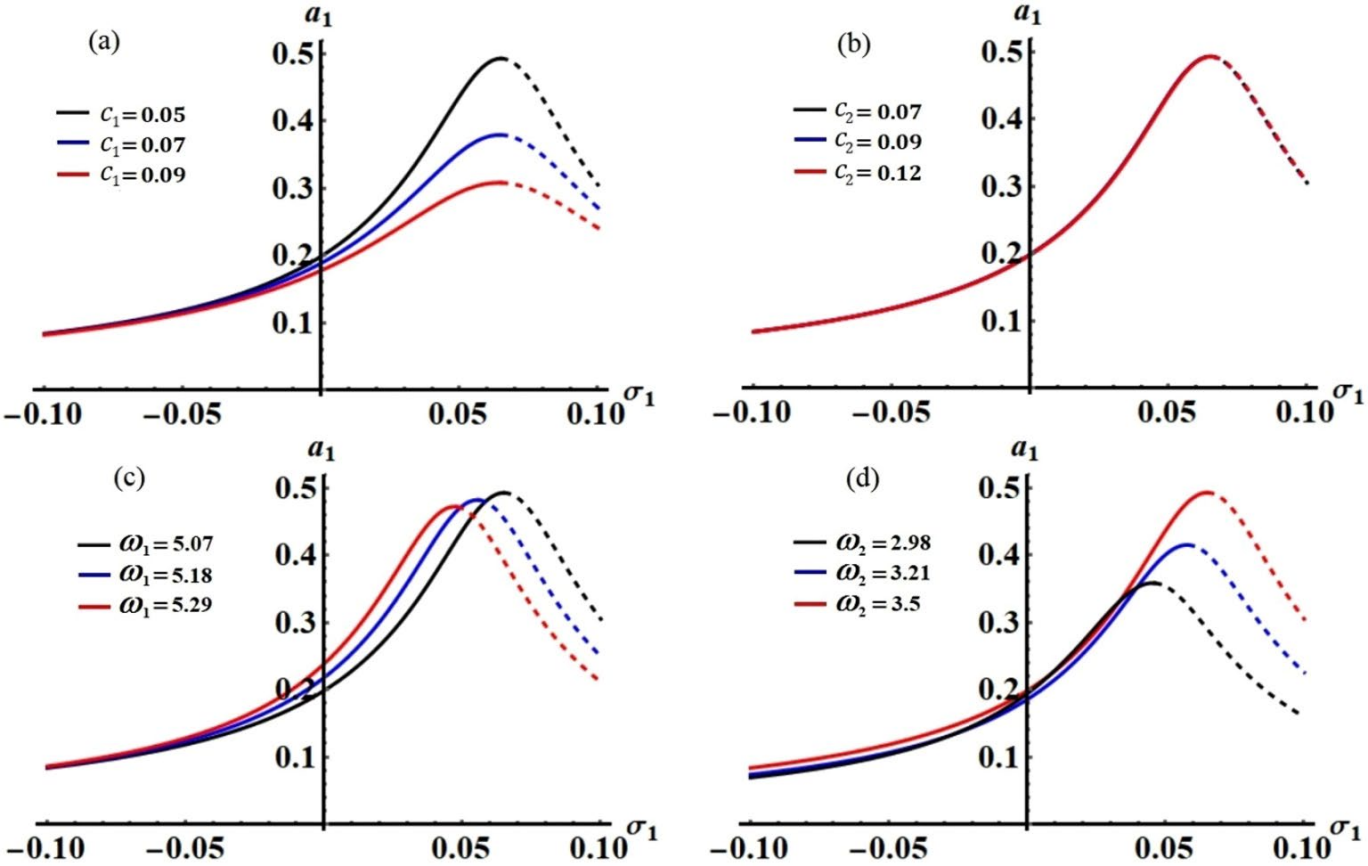
The FRC in the plane $\sigma_{1} a_{1}$ at: (**a**) $c_{1} ( = 0.05,\,0.07,\,0.09),$ (**b**) $c_{2} ( = 0.07,\,0.09,\,0.12),$ (**c**) $\omega_{1} ( = 5.07,\,5.18,\,5.29),$ and (**d**) $\omega_{2} ( = 2.98,\,3.21,\,3.5)$**.**

**Figure 12:**
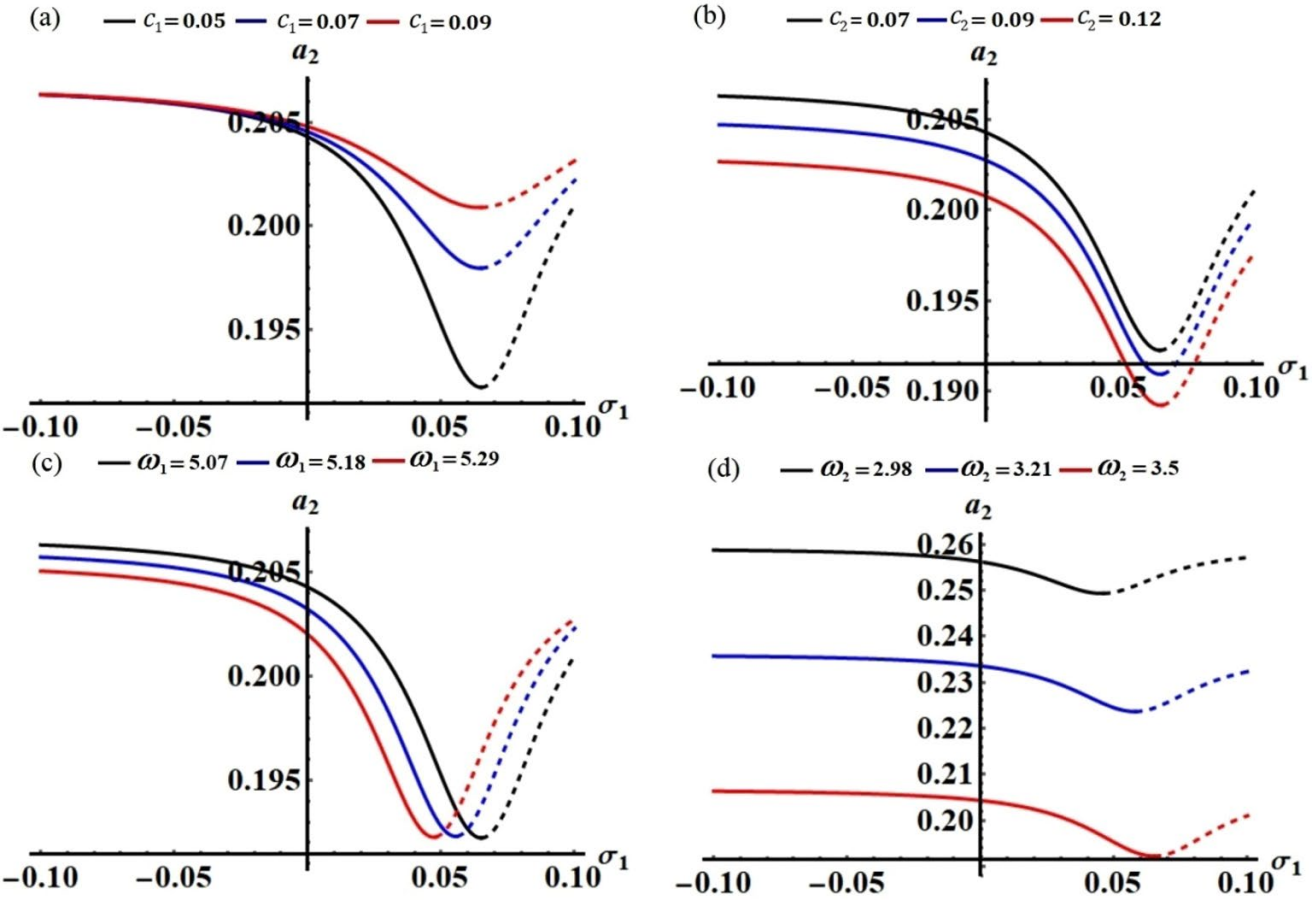
The FRC in the plane $\sigma_{1} a_{2}$ at: (**a**) $c_{1} ( = 0.05,\,0.07,\,0.09),$ (**b**) $c_{2} ( = 0.07,\,0.09,\,0.12),$ (**c**) $\omega_{1} ( = 5.07,\,5.18,\,5.29),$ and (**d**) $\omega_{2} ( = 2.98,\,3.21,\,3.5)$**.**

Figures 11 and 12 have been drawn, respectively, in planes $\sigma_{1} a_{1}$ and $\sigma_{1} a_{2}$ to represent the frequency response curves (FRC) when $c_{j}$ and $\omega_{j}$ have different values in addition to the value of the detuning parameter $\sigma_{2}$ which is computed according to the relation $\sigma_{2} = p_{2} - \omega$. In more details, curves of Figs. 11a and 12a summarise the effect of the different values of $c_{1} ( = 0.05,\,0.07,\,0.09),$ on the generated curves. Examining of these figures reveal that each curve contains only one critical fixed point over the whole domain as tabulated in Table 1. The stability and instability zones are discovered in the ranges $\sigma_{1} < 0.065$, and $0.065 \le \sigma_{1}$, respectively. It is critical to note that the solid curves represent the range of stable fixed points, whereas the dashed lines depict the range of unstable fixed points.

**Table 1:** Critical and peak fixed points for the curves of Figs. 11 and 12 when $\sigma_{2} = p_{2} - \omega$.

Figure	Critical points	Peaks points	$\sigma_{2} = p_{2} - \omega$
Figure 11a	(0.065, 0.493), (0.065, 0.379), (0.065, 0.308)	**–**	$\sigma_{2} = - 0.2169$
Figure 11b	(0.065, 0.493), (0.066, 0.4928)	**–**	$\sigma_{2} = - 0.2169$
Figure 11c	(0.065, 0.493), (0.056, 0.482), (0.048, 0.4722)	**–**	$\sigma_{2} ( = - 0.2169,\, - 0.2122,\,0.2078)$
Figure 11d	(0.065, 0.493), (0.058, 0.415), (0.046, 0.358)	**–**	$\sigma_{2} ( = - 0.2169,\, - 0.16,\, - 0.1153)$
Figure 12a	(0.065, 0.192), (0.065, 0.2009)	**–**	$\sigma_{2} = - 0.2169$
Figure 12b	(0.065, 0.192), (0.066, 0.1909), (0.066, 0.189)	**–**	$\sigma_{2} = - 0.2169$
Figure 12c	(0.065, 0.192), (0.056, 0.1923), (0.048, 0.1922)	**–**	$\sigma_{2} ( = - 0.2169,\, - 0.2122,\,0.2078)$
Figure 12d	(0.065, 0.192), (0.058, 0.2236), (0.046, 0.2492)	**–**	$\sigma_{2} ( = - 0.2169,\, - 0.16,\, - 0.1153)$

According to the positive impact of the various values of the other damping parameter $c_{2} ( = 0.07,\,0.09,\,0.12),$ Figs. 11b and 12b are drawn to display the FRC at these values. As aforementioned, one critical fixed point is observed for each curve, in which stable and instable fixed points at $c_{2} = 0.07$ are generated, respectively, in the ranges $\sigma_{1} < 0.065$ and $0.065 \le \sigma_{1}$. Whereas, at $c_{2} ( = 0.09,\,0.12)$ one finds other regions of stability and instability at the ranges $\sigma_{1} \le 0.066$ and $0.066 < \sigma_{1}$. The drawn FRC in Figs. 11c and 12c show the good influence of various values of the frequency $\omega_{1} ( = 5.07,\,5.18,\,5.29)$ on the behavior of the stability and instability areas, in which there exists a single fixed point for each curve. It is observed that the areas of stability are found in the ranges $\sigma_{1} \le 0.065,$ $\sigma_{1} \le 0.056,$ and $\sigma_{1} \le 0.048$, while the instability areas of the fixed points are generated in the range $0.065 < \sigma_{1} ,$ $0.056 \le \sigma_{1} ,$ and $0.048 \le \sigma_{1}$. Other stability and instability regions have been plotted at different values of $\omega_{2} ( = 2.98,\,3.21,\,3.5)$ as seen in Figs. 11d and 12d. The stable fixed points are found in the ranges $\sigma_{1} \le 0.046,$ $\sigma_{1} \le 0.058,$ and $\sigma_{1} \le 0.065,$ while the unstable ones occurs in the ranges $0.046 < \sigma_{1} ,$ $0.058 < \sigma_{1} ,$ and $0.065 < \sigma_{1}$.

**Figure 13:**
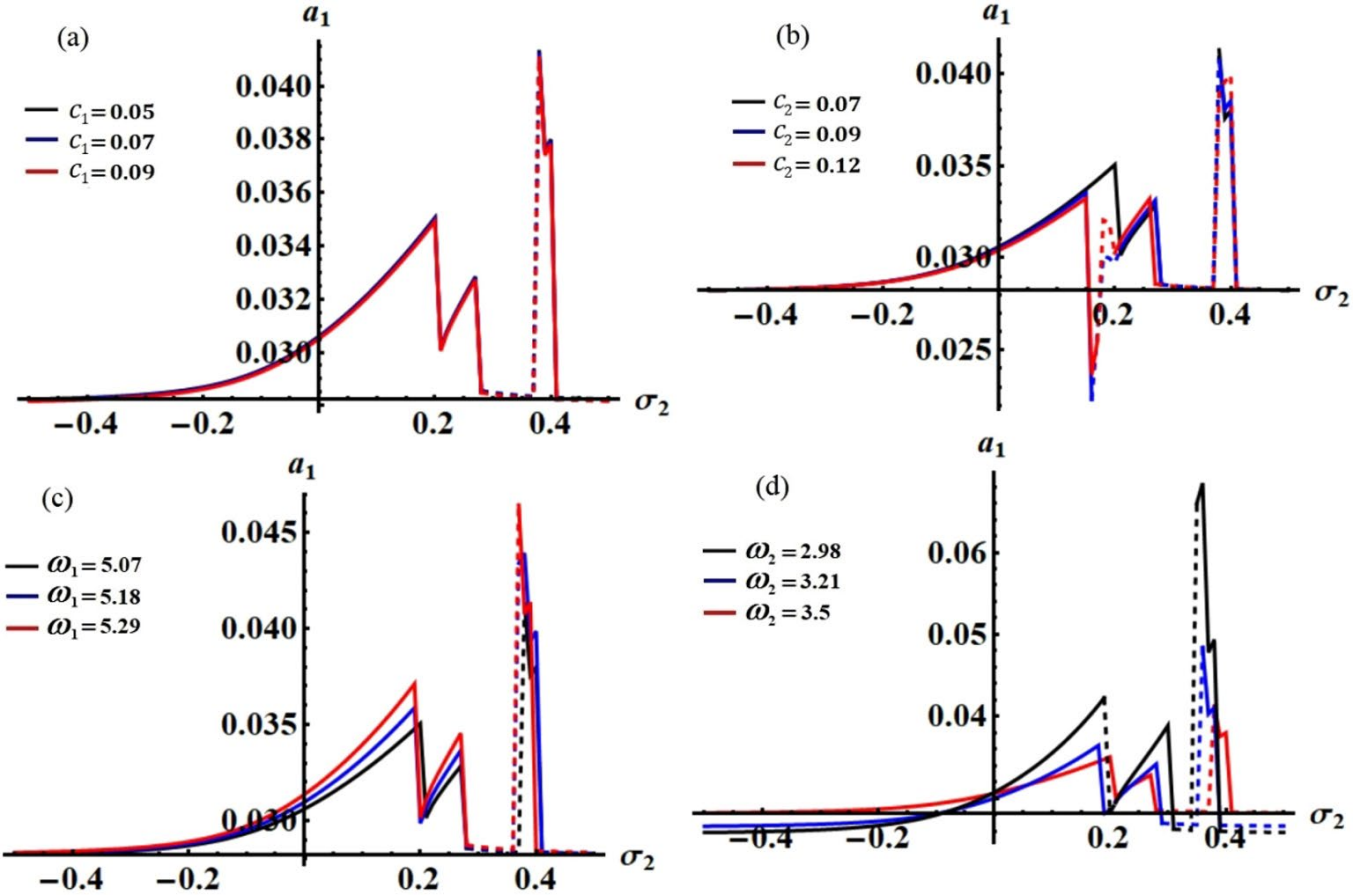
The FRC in the plane $\sigma_{2} a_{1}$ at: (**a**) $c_{1} ( = 0.05,\,0.07,\,0.09),$ (**b**) $c_{2} ( = 0.07,\,0.09,\,0.12),$ (**c**) $\omega_{1} ( = 5.07,\,5.18,\,5.29),$ and (**d**) $\omega_{2} ( = 2.98,\,3.21,\,3.5)$**.**

**Figure 14:**
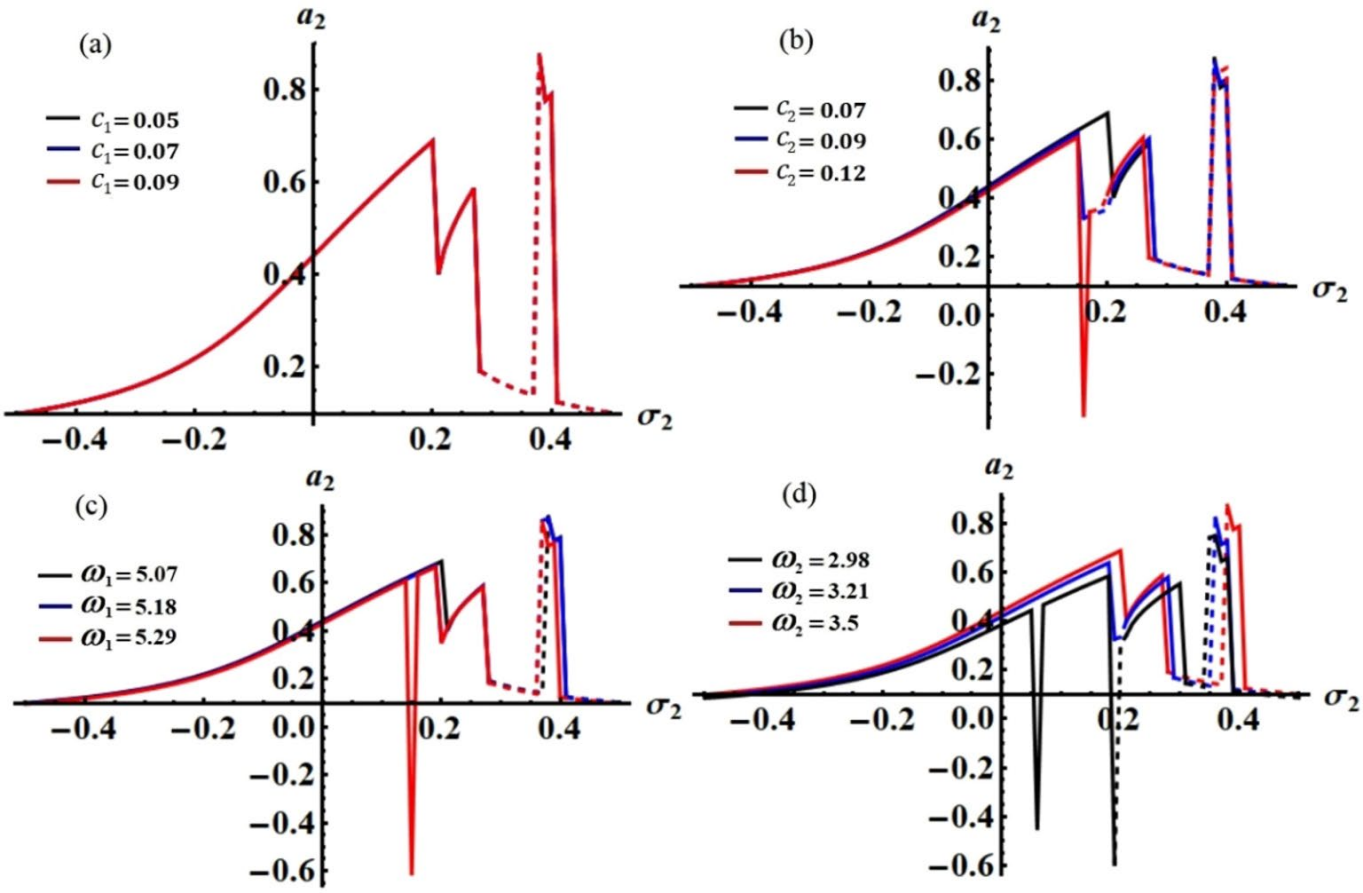
The FRC in the plane $\sigma_{2} a_{2}$ at: (**a**) $c_{1} ( = 0.05,\,0.07,\,0.09),$ (**b**) $c_{2} ( = 0.07,\,0.09,\,0.12),$ (**c**) $\omega_{1} ( = 5.07,\,5.18,\,5.29),$ and (**d**) $\omega_{2} ( = 2.98,\,3.21,\,3.5)$**.**

According to the value of the detuning parameter $\sigma_{1}$, which is calculated using the relation $\sigma_{1} = p_{1} - 1$, Figs. 13 and 14 have been drawn, respectively, to depict the FRC in planes $\sigma_{2} a_{1}$ and $\sigma_{2} a_{2}$ when $c_{j} \,\,(j = 1,2)$ and $\omega_{j}$ have various values. The range of stable fixed points is shown by the solid lines, while the range of unstable ones is shown by the dashed lines. These figures illustrate that each curve contains critical and peak fixed points, which are tabulated in Tables 2 and 3.

**Table 2:** Critical and peak fixed points for the curves of Fig. 13 when $\sigma_{1} = p_{1} - 1$.

Figure	Critical points	Peaks points	$\sigma_{1} = p_{1} - 1$
Figure 13a	(0.28, 0.0285), (0.38, 0.0412), (0.41, 0.0283)	(0.2007, 0.03502), (0.2109, 0.03016), (0.2711, 0.03279), (0.3698, 0.02837), (0.3903, 0.03759), (0.4016, 0.03794), (0.3709, 0.02827), (0.3898, 0.03756)	$\sigma_{1} = - 0.4204$
Figure 13b	(0.28, 0.0285), (0.38, 0.04128), (0.41, 0.0283), (0.16, 0.0223), (0.21, 0.0305), (0.17, 0.0255), (0.2, 0.0302), (0.27, 0.02858)	(0.2007, 0.03502), (0.2109, 0.03016), (0.2711, 0.03279), (0.3698, 0.02837), (0.3903, 0.03759), (0.4016, 0.03794), (0.1513,0.03352), (0.1815, 0.0301), (0.2008, 0.0297), (0.3699, 0.02838), (0.1601, 0.02373), (0.2606, 0.03326), (0.3707, 0.02833), (0.3804, 0.03913), (0.4107, 0.02826)	$\sigma_{1} = - 0.4204$
Figure 13c	(0.28, 0.02856), (0.38, 0.04128), (0.41, 0.0283), (0.37, 0.0434), (0.4, 0.0284)	(0.2007, 0.03502), (0.2109, 0.03016), (0.2711, 0.03279), (0.3698, 0.02837), (0.3903, 0.03759), (0.4016, 0.03794), (0.3895, 0.03935), (0.1904, 0.03713), (0.2701, 0.3446), (0.3799, 0.04077)	$$\begin{gathered} \sigma_{1} ( = - 0.4204,\, - 0.4077, \hfill \\ \,\,\,\,\,\,\, - 0.3952) \hfill \\ \end{gathered}$$
Figure 13d	(0.28, 0.0285), (0.38, 0.0412), (0.41, 0.0283), (0.19, 0.02816), (0.21, 0.02966), (0.29, 0.02697), (0.36, 0.0484), (0.39, 0.0267), (0.19, 0.04226), (0.31, 0.02625), (0.35, 0.066)	(0.2007, 0.03502), (0.2109, 0.03016), (0.2711, 0.03279), (0.3698, 0.02837), (0.3903, 0.03759), (0.4016, 0.03794), (0.1805, 0.03649), (0.2806, 0.03412), (0.3518, 0.02672), (0.3707, 0.04031), (0.36, 0.06836), (0.3804, 0.04929)	$$\begin{gathered} \sigma_{1} ( = - 0.4204,\, - 0.3684, \hfill \\ \,\,\,\,\,\,\, - 0.3204) \hfill \\ \end{gathered}$$

**Table 3:** Critical and peaks fixed points for the curves of Fig. 14 when $\sigma_{1} = p_{1} - 1$.

Figure	Critical points	Peaks points	$\sigma_{1} = p_{1} - 1$
Figure 14a	(0.28, 0.1922), (0.38, 0.874), (0.41, 0.1248)	(0.2018, 0.6877), (0.2107, 0.4039), (0.2692, 0.5833), (0.3708, 0.1396), (0.3912, 0.7758), (0.4013, 0.7889)	$\sigma_{1} = - 0.4204$
Figure 14b	(0.28, 0.1922), (0.38, 0.874), (0.41, 0.1248), (0.16, 0.331638), (0.21, 0.43259), (0.17, 0.352582), (0.2, 0.412032), (0.27, 0.19655)	(0.2018, 0.6877), (0.2107, 0.4039), (0.2692, 0.5833), (0.3708, 0.1396), (0.3912, 0.7758), (0.4013, 0.7889), (0.1509, 0.6214), (0.2005, 0.3638), (0.2704, 0.5986), (0.1601, − 0.3443), (0.3817, 0.8191), (0.4006, 0.8377), (0.4112, 0.1225)	$\sigma_{1} = - 0.4204$
Figure 14c	(0.28, 0.1922), (0.38, 0.874), (0.41, 0.1248), (0.37, 0.858698), (0.4, 0.122753)	(0.2018, 0.6877), (0.2107, 0.4039), (0.2692, 0.5833), (0.3708, 0.1396), (0.3912, 0.7758), (0.4013, 0.7889), (0.19, 0.6715), (0.1997, 0.3525), (0.2711, 0.5831), (0.3597, 0.1385), (0.3695, 0.8565), (0.3803, 0.8675), (0.1406, 0.6064), (0.1501, − 0.6175), (0.1595, 0.6318), (0.1899, 0.6642), (0.2013, 0.3524), (0.3606, 0.1376), (0.3814, 0.7519), (0.3909, 0.7635)	$$\begin{gathered} \sigma_{1} ( = - 0.4204,\, - 0.4077,\, \hfill \\ \,\,\,\,\,\,\, - 0.3952) \hfill \\ \end{gathered}$$
Figure 14d	(0.28, 0.1922), (0.38, 0.874), (0.41, 0.1248), (0.19, 0.327095), (0.21, 0.400484), (0.29, 0.168595), (0.36, 0.821827), (0.39, 0.120887), (0.19, − 0.59488), (0.31, 0.1454), (0.35, 0.742534)	(0.2018, 0.6877), (0.2107, 0.4039), (0.2692, 0.5833), (0.3708, 0.1396), (0.3912, 0.7758), (0.4013, 0.7889), (0.1802, 0.6387), (0.2806, 0.5799), (0.3505, 0.1346), (0.3708, 0.7136), (0.3809, 0.7271), (0.05086, 0.4416), (0.05956, − 0.4534), (0.06935,0.4658), (0.1814, 0.5847), (0.3, 0.558), (0.3402, 0.1311), (0.3609, 0.752), (0.3718, 0.06453), (0.3816, 0.6574)	$$\begin{gathered} \sigma_{1} ( = - 0.4204,\, - 0.3684,\, \hfill \\ \,\,\,\,\,\,\, - 0.3204) \hfill \\ \end{gathered}$$

The curves in Figs. 13a and 14a point out the effect of the damping parameter $$c_{1}$$ at different values $$c_{1} ( = 0.05,\,0.07,\,0.09)$$. These figures illustrate that each curve contains three critical fixed points over the whole domain. The stability zones are identified in the ranges $$\sigma_{2} < 0.28$$, and $$0.38 \le \sigma_{2} < 0.41$$. Whereas, the instability zones are found in the ranges $$0.28 \le \sigma_{2} < 0.38$$, and $$0.41 \le \sigma_{2}$$. On the other hand, Figs. 13b and 14b show the FRC for various values of the damping parameter $$c_{2} ( = 0.07,\,0.09,\,0.12)$$. Three critical fixed points are observed in the graphed curves at $$c_{2} ( = 0.07,\,0.12)$$. The stability zones at $$c_{2} = 0.07$$ are found in the ranges $$\sigma_{2} < 0.28$$ and $$0.38 \le \sigma_{2} < 0.41$$. While, the instability zones at $$c_{2} = 0.07$$ are found in the ranges $$0.28 \le \sigma_{2} < 0.38$$ and $$0.41 \le \sigma_{2}$$. At $$c_{2} = 0.12,$$ the ranges $$\sigma_{2} < 0.17$$ and $$0.2 \le \sigma_{2} < 0.27$$ indicate the stability zones, whereas $$0.17 \le \sigma_{2} < 0.2$$ and $$0.2 \le \sigma_{2}$$ express the instability ones. In addition, at $$c_{2} = 0.09$$ the stability ranges are $$\sigma_{2} < 0.16,$$$$0.21 \le \sigma_{2} < 0.28,$$ and $$0.38 \le \sigma_{2} < 0.41$$. As well as, the instability ranges are $$0.16 \le \sigma_{2} < 0.21,$$ $$0.28 \le \sigma_{2} < 0.38,$$ and $$0.41 \le \sigma_{2}$$. That is, each curve at $$c_{2} = 0.09$$ contains five critical fixed points over the whole domain. Figures 13c and 14c show the effect of various values of the frequency $$\omega_{1} ( = 5.07,\,5.18,\,5.29)$$ on the behavior of the stability and instability areas, in which there are three fixed points for each curve. It is observed that the areas of stability are generated at $$\omega_{1} = 5.07$$ in the ranges $$\sigma_{2} < 0.28$$ and $$0.38 \le \sigma_{2} < 0.41$$ while at $$\omega_{1} = 5.18,$$ they will be $$\sigma_{2} < 0.28$$ and $$0.37 \le \sigma_{2} < 0.41$$, whereas the stability regions at at $$\omega_{1} = 5.29$$ are $$\sigma_{2} < 0.28$$ and $$0.37 \le \sigma_{2} < 0.4$$. On the other hand, their related instability areas at $$\omega_{1} = 5.07,$$ at $$\omega_{1} = 5.18,$$ and $$\omega_{1} = 5.29$$, are discovered in the ranges $$(\sigma_{2} < 0.38,\,\,\,0.41 \le \sigma_{2} \le 0.5),$$ $$(0.28 \le \sigma_{2} < 0.37,\,\,0.41 \le \sigma_{2} ),$$ and $$(0.28 \le \sigma_{2} < 0.37,\,\,0.4 \le \sigma_{2} )$$, respectively. The stability and instability zones at different values of $$\omega_{2} ( = 2.98,\,3.21,\,3.5)$$ are portrayed in Figs. 13d and 14d. At $$\omega_{2} = 2.98,$$ the stable fixed points are found in the ranges $$\sigma_{2} < 0.19,$$ $$0.21 \le \sigma_{2} < 0.31,$$ and $$0.35 \le \sigma_{2} < 0.39,$$ while the unstable ones occurs in the ranges $$0.19 \le \sigma_{2} < 0.21,$$ $$0.31 \le \sigma_{2} < 0.35,$$ and $$0.39 \le \sigma_{2}$$. At $$\omega_{2} = 3.21,$$ the stability regions are found in the ranges $$\sigma_{2} < 0.19,$$ $$0.21 \le \sigma_{2} < 0.29,$$ and $$0.36 \le \sigma_{2} < 0.39,$$ while the unstable ones are observed in the ranges $$0.19 \le \sigma_{2} < 0.21,$$ $$0.29 \le \sigma_{2} < 0.36,$$ and $$0.39 \le \sigma_{2}$$. Finally, at $$\omega_{2} = 3.5,$$ the stability areas are found in the ranges $$\sigma_{2} < 0.28,$$ and $$0.38 \le \sigma_{2} < 0.41,$$ while the unstable ones are given in the ranges $$0.28 \le \sigma_{2} < 0.38$$ and $$0.41 \le \sigma_{2}$$.

## Non-linear analysis

The purpose of this section is to clarify the properties of the non-linear amplitudes of the system of Eqs. (33)–(36) and look into its stability. Consequently, we take into account the below transformation^39,40^

50 $$ D_{j} = [U_{j} (\tau_{2} ) + i\,V_{j} (\tau_{2} )]\,e^{{i\tilde{\sigma }_{j} \tau_{2} }} \,\,\,\,(j = 1,2), $$

where $U_{j}$ and $V_{j}$ are, respectively, the amplitudes’ real and imaginary components.

Separating the distinct parts that yield from the substitution of (50) into (33)–(36) to obtain

51 $$ \frac{{dv_{1} }}{d\tau } = - \frac{{f_{1} }}{4} - \frac{{c_{1} v_{1} }}{2} + u_{1} \left[ {\frac{3}{2}\alpha {\kern 1pt} \Re_{r}^{2} {\kern 1pt} - \sigma_{1} + \frac{{3\omega^{2} (\omega^{2} - 1)(u_{2}^{2} + v_{2}^{2} )}}{{4\omega^{2} - 1}}} \right], $$

52 $$ \frac{{du_{1} }}{d\tau } = - \frac{1}{2}c_{1} u_{1} + v_{1} \left[ {\sigma_{1} - \frac{3}{2}\alpha {\kern 1pt} \Re_{r}^{2} - \frac{{3\omega^{2} (\omega^{2} - 1)(u_{2}^{2} + v_{2}^{2} )}}{{4\omega^{2} - 1}}} \right], $$

53 $$ \frac{{dv_{2} }}{d\tau } = - \frac{1}{4\omega }f_{2} - \frac{1}{2}{\kern 1pt} c_{2} {\kern 1pt} v_{2} + u_{2} \left\{ { - \sigma_{2} + \frac{\omega }{{4(4\omega^{2} - 1)}}\left[ {2(2\omega - 1)(\omega^{2} + 2)(u_{1}^{2} + v_{1}^{2} ) - (3\omega^{2} + 1)(8\omega^{2} + 1)(u_{2}^{2} + v_{2}^{2} )} \right]} \right\}, $$

54 $$ \frac{{du_{2} }}{d\tau } = - \frac{1}{2}{\kern 1pt} c_{2} {\kern 1pt} u_{2} + v_{2} \left\{ {\sigma_{2} - \frac{\omega }{{4(4\omega^{2} - 1)}}\left[ {2(2\omega - 1)(\omega^{2} + 2)(u_{1}^{2} + v_{1}^{2} ) + (3\omega^{2} + 1)(8\omega^{2} + 1)(u_{2}^{2} + v_{2}^{2} )} \right]} \right\}, $$

where

$$ U_{j} = \varepsilon \,u_{j} ,\,\,\,\,\,\,\,\,V_{j} = \varepsilon \,v_{j} . $$

**Figure 15:**
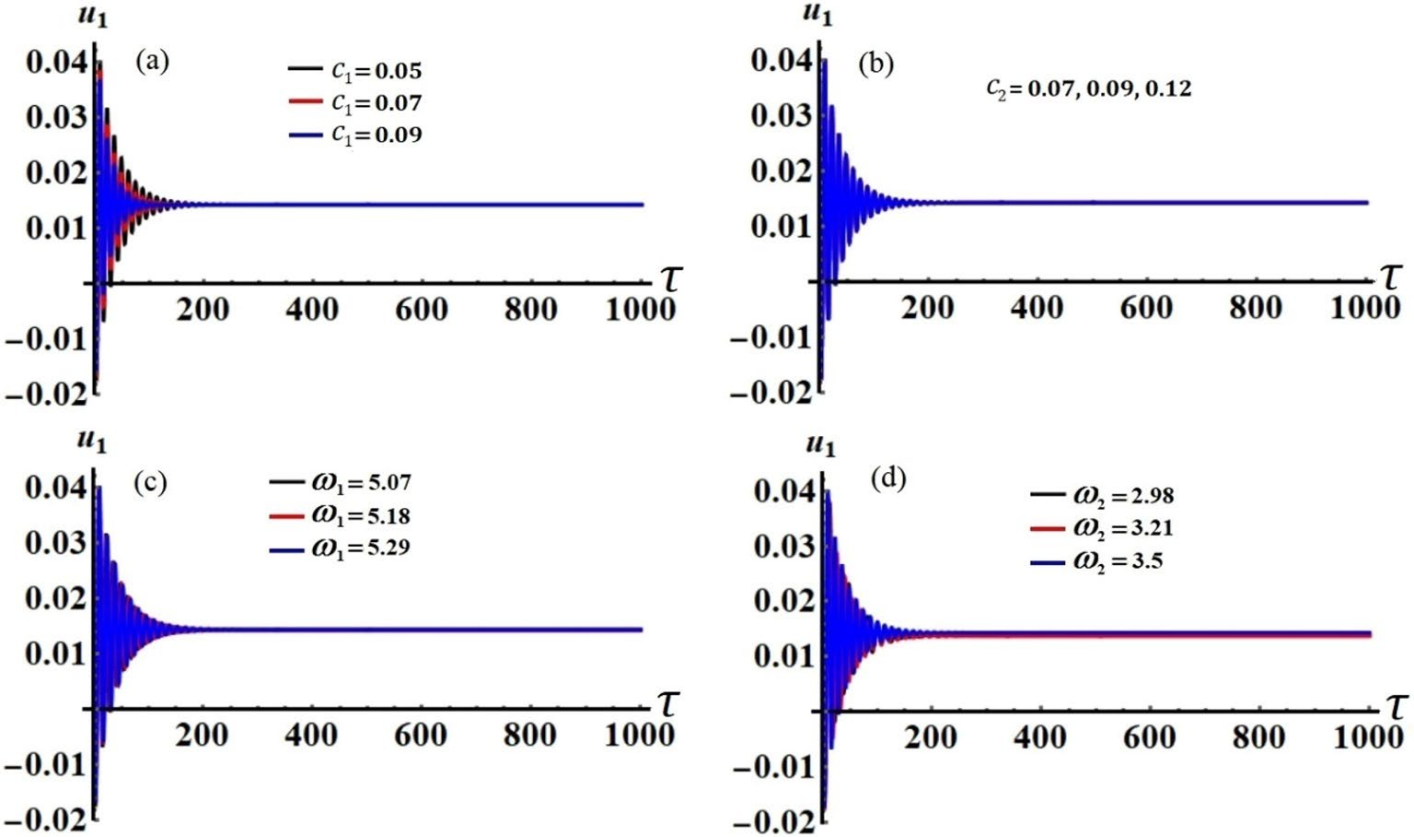
The modified amplitude $u_{1}$ via time $\tau$ when: (**a**) $c_{1} ( = 0.05,\,0.07,\,0.09),$ (**b**) $c_{2} ( = 0.07,\,0.09,\,0.12),$ (**c**) $\omega_{1} ( = 5.07,\,5.18,\,5.29),$ and (**d**) $\omega_{2} ( = 2.98,\,3.21,\,3.5)$.

**Figure 16:**
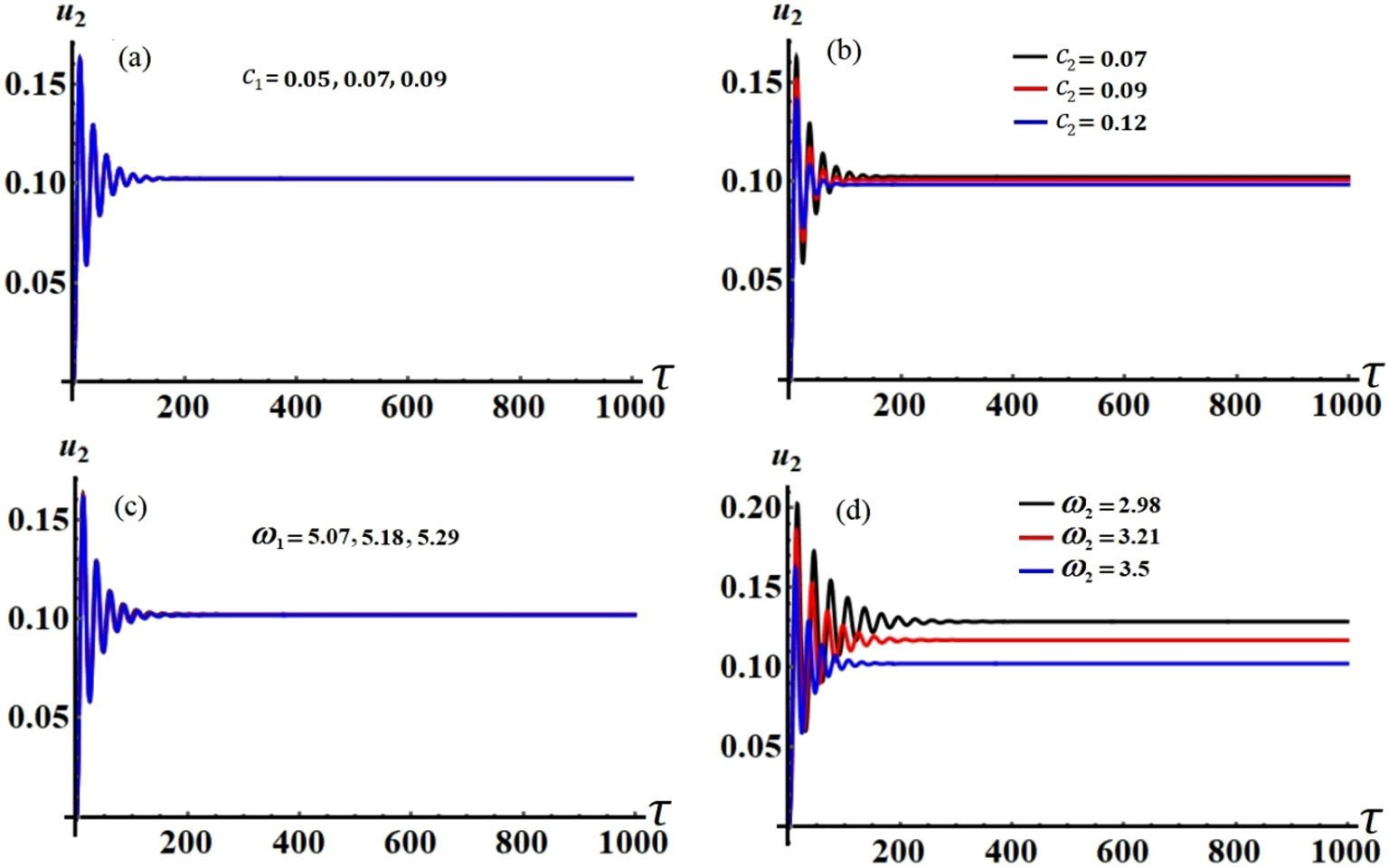
The modified amplitude $u_{2}$ via time $\tau$ when: (**a**) $c_{1} ( = 0.05,\,0.07,\,0.09),$ (**b**) $c_{2} ( = 0.07,\,0.09,\,0.12),$ (**c**) $\omega_{1} ( = 5.07,\,5.18,\,5.29),$ and (**d**) $\omega_{2} ( = 2.98,\,3.21,\,3.5)$.

**Figure 17:**
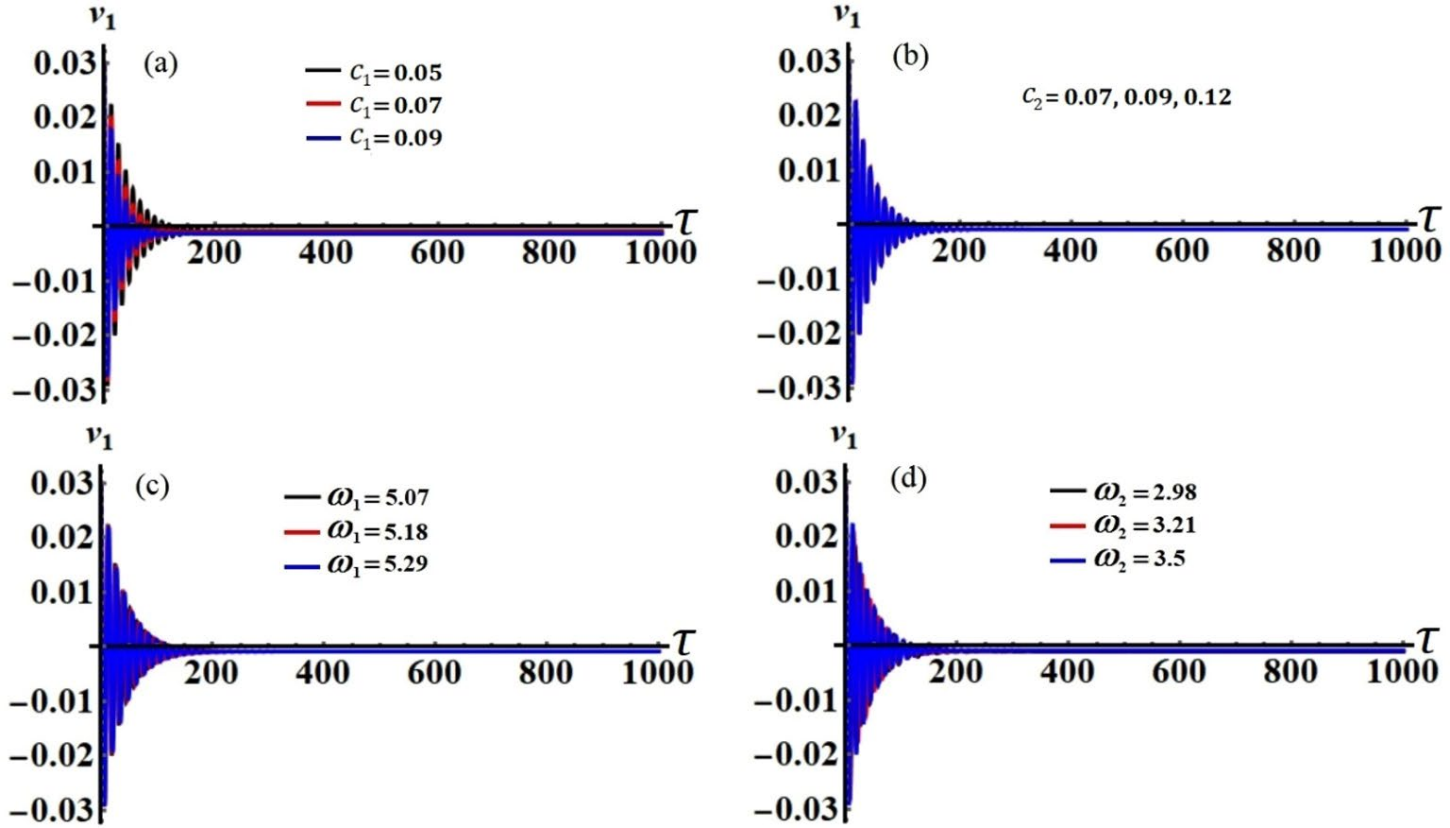
The modified amplitude $v_{1}$ via time $\tau$ when: (**a**) $c_{1} ( = 0.05,\,0.07,\,0.09),$ (**b**) $c_{2} ( = 0.07,\,0.09,\,0.12),$ (**c**) $\omega_{1} ( = 5.07,\,5.18,\,5.29),$ and (**d**) $\omega_{2} ( = 2.98,\,3.21,\,3.5)$.

**Figure 18:**
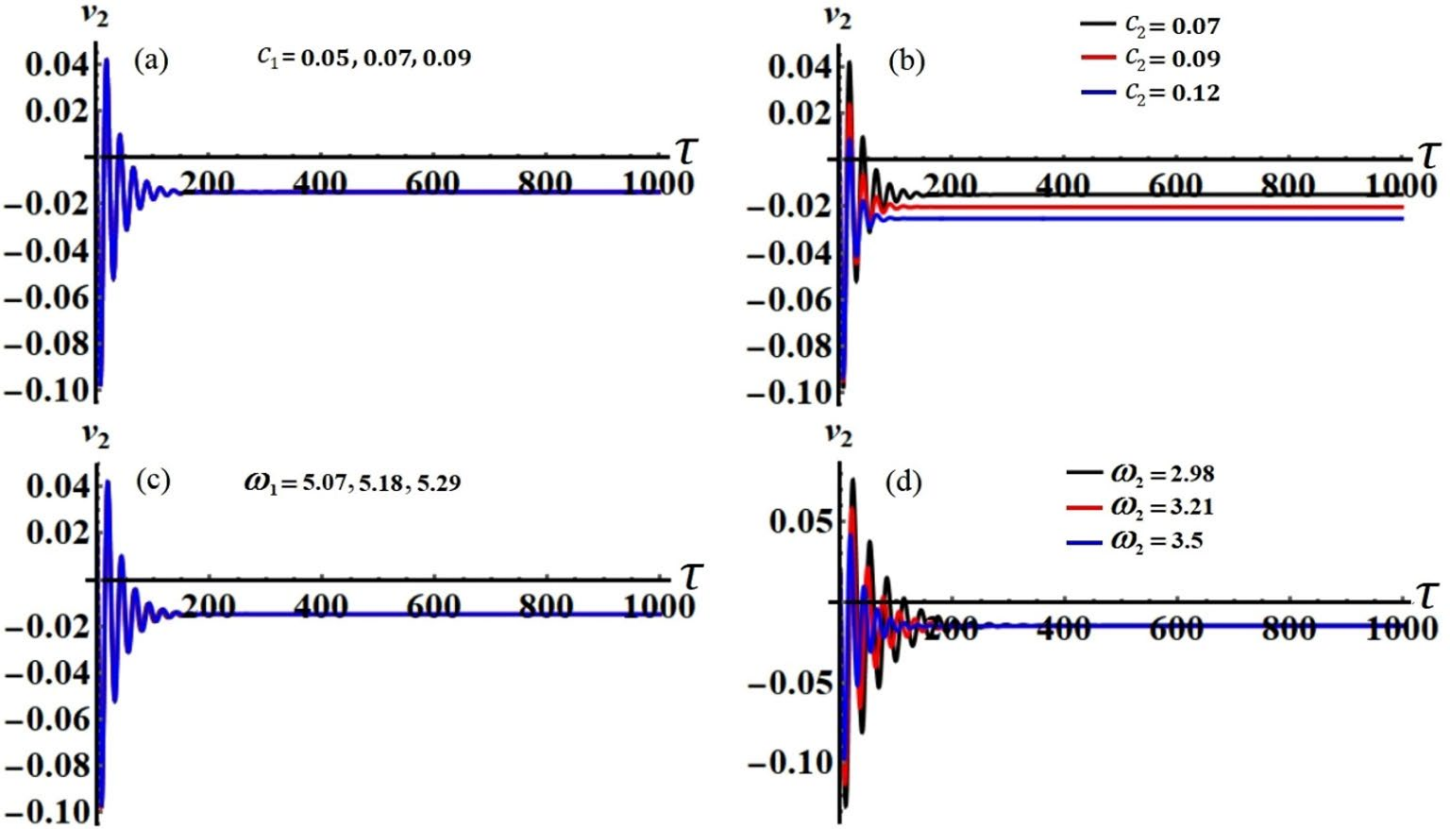
The modified amplitude $v_{2}$ via time $\tau$ when: (**a**) $c_{1} ( = 0.05,\,0.07,\,0.09),$ (**b**) $c_{2} ( = 0.07,\,0.09,\,0.12),$ (**c**) $\omega_{1} ( = 5.07,\,5.18,\,5.29),$ and (**d**) $\omega_{2} ( = 2.98,\,3.21,\,3.5)$.

The modified amplitudes have been justified over an entire period of time in distinct parametric zones based on the previously mentioned data of the used parameters, in which their properties may be displayed in the curves of phase plane, as shown in Figs. 13, 14, 15, 16, 17 and 18.

**Figure 19:**
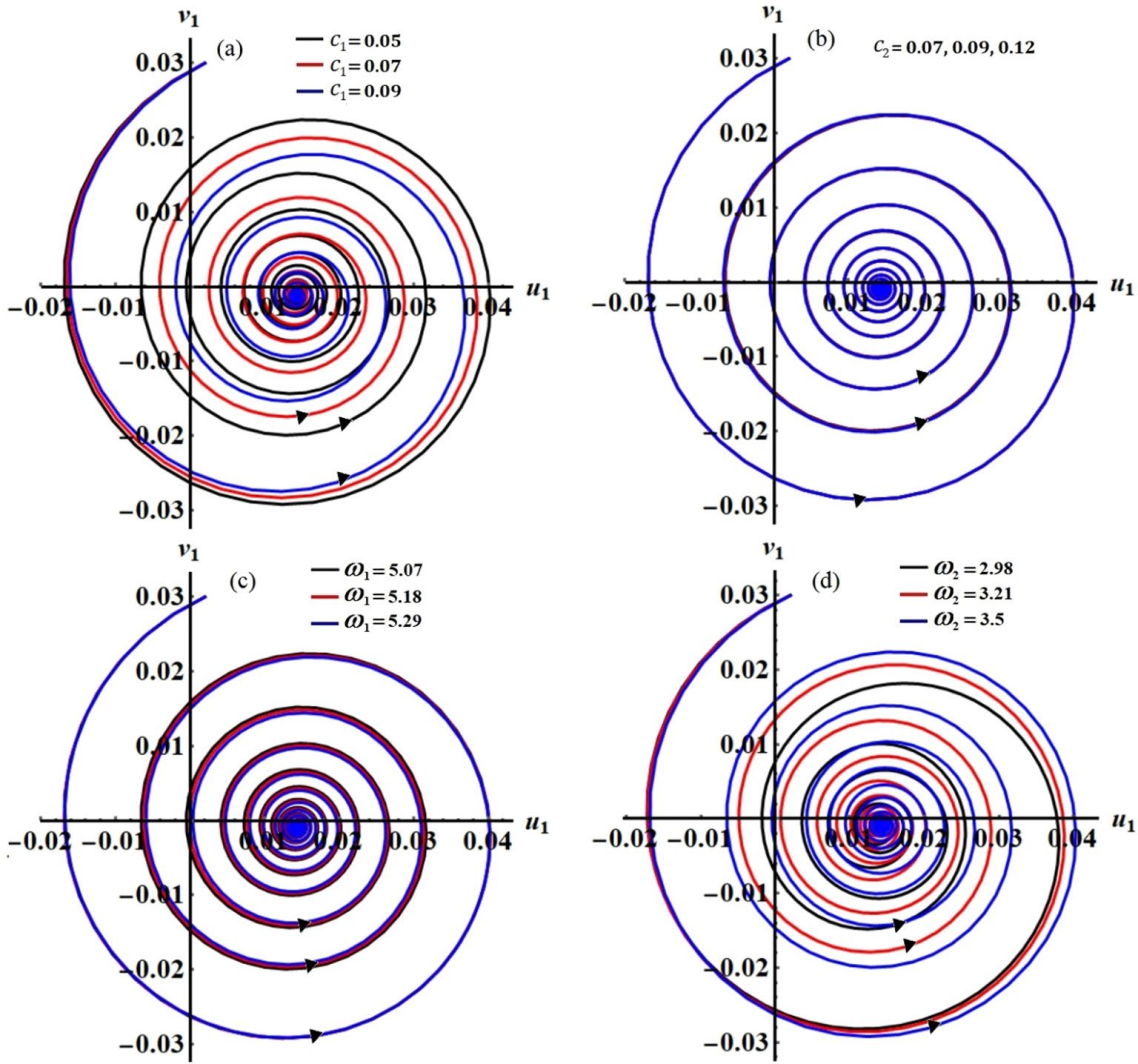
The projection of amplitudes’ paths $u_{1}$ and $v_{1}$ in plane $u_{1} v_{1}$ when: (**a**) $c_{1} ( = 0.05,\,0.07,\,0.09),$ (**b**) $c_{2} ( = 0.07,\,0.09,\,0.12),$ (**c**) $\omega_{1} ( = 5.07,\,5.18,\,5.29),$ and (**d**) $\omega_{2} ( = 2.98,\,3.21,\,3.5)$.

**Figure 20:**
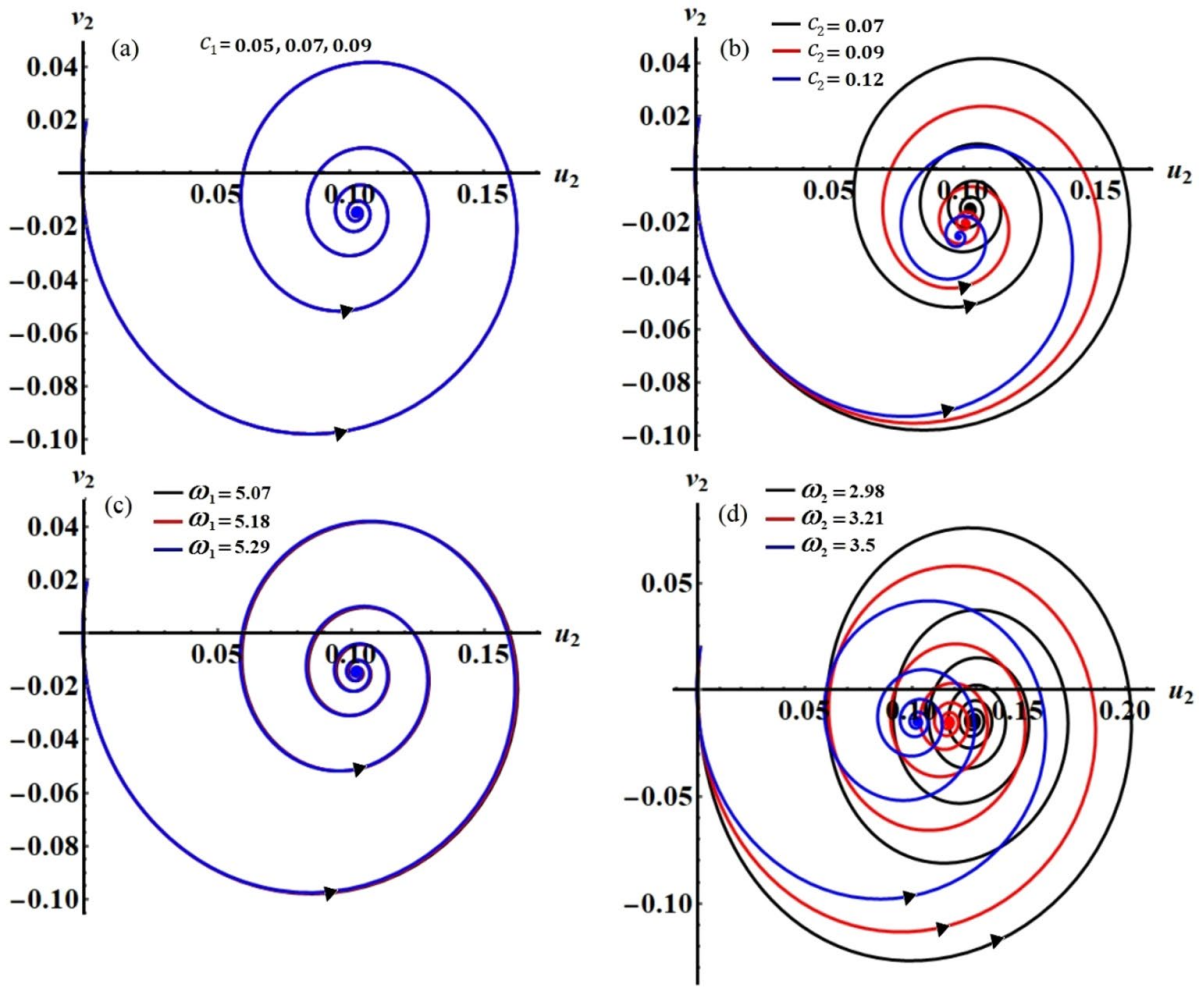
The projection of amplitudes’ paths $u_{2}$ and $v_{2}$ in plane $u_{2} v_{2}$ when: (**a**) $c_{1} ( = 0.05,\,0.07,\,0.09),$ (**b**) $c_{2} ( = 0.07,\,0.09,\,0.12),$ (**c**) $\omega_{1} ( = 5.07,\,5.18,\,5.29),$ and (**d**) $\omega_{2} ( = 2.98,\,3.21,\,3.5)$.

Figures 15, 16, 17 and 18 show how the new modified amplitudes $u_{j}$ and $v_{j}$ change over time $\tau$ according to the numerical solutions for the system of Eqs. (51)–(54) when $c_{j}$ and $\omega_{j}$ have different values. Decay waves have been graphed in light of these values until they become nearly motionless at the end of the time period. It is noted that these curves behave in a stable manner, which can be asserted when the projections of these curves are plotted in a suitable phase plane. Therefore, curves in Figs. 19 and 20 have been drawn to explore how the projections of $u_{j}$ and $v_{j}$ are plotted in the planes $u_{j} v_{j}$ when the aforementioned values of $c_{j}$ and $\omega_{j}$ are considered. The behavior of the resulting curves shows spiral patterns oriented to one point for each curve, indicating that this behavior is steady and free of chaos.

## Conclusion

This work has focused on analysing the planar movement of a spring pendulum with two degrees of freedom that undergoes vibrations, in which its pivot point is confined to move along a trajectory that resembles a Lissajous curve. By utilizing the system’s coordinates, the EOM for the system have been successfully derived using Lagrange’s equations. The AMS technique has been utilized to obtain highly refined solutions for this system, surpassing previous approximations. These solutions have been contrasted with the obtained NS through the 4RKA method to reveal the exceptional precision achieved with the employed perturbation approach. The classification of resonance situations and the development of ME have been accomplished, taking into account the solvability constraints. Therefore, the solutions for steady-state scenarios have been verified. The RHC has been utilized to evaluate and plot both stable and unstable regions. The obtained outcomes, including FRC and stability zones, are displayed and visually depicted to evaluate the beneficial impact of various physical parameter values on the behavior of the analysed system. Upon scrutinizing the stability and instability zones, it becomes evident that the behavior of the system remains stable for a significant portion of its parameters. Furthermore, the nonlinear stability analysis of the adjusted amplitudes has been examined to reveal their stationary behavior.

## Data Availability

All data generated or analysed during this study are included in this published article.
